# Traffic jam regulates the function of the ovarian germline stem cell progeny differentiation niche during pre-adult stage in *Drosophila*

**DOI:** 10.1038/s41598-019-45317-6

**Published:** 2019-07-12

**Authors:** Mengjie Li, Xiaolong Hu, Shu Zhang, Margaret S. Ho, Geng Wu, Lei Zhang

**Affiliations:** 1 State Key Laboratory of Microbial Metabolism, School of Life Sciences & Biotechnology, The Joint International Research Laboratory of Metabolic & Developmental Sciences, Shanghai Jiao Tong University, Shanghai, 200240 China; 2 School of Life Sciences and Biotechnology, Shanghai Jiao Tong University, Shanghai, 200240 China; 3 State Key Laboratory of Cell Biology, CAS Center for Excellence in Molecular Cell Science, Innovation Center for Cell Signaling Network, Shanghai Institute of Biochemistry and Cell Biology, Chinese Academy of Sciences, University of Chinese Academy of Sciences, Shanghai, 200031 China; 4 School of Life Science and Technology, Shanghai Tech University, Shanghai, 201210 China

**Keywords:** Stem-cell niche, Development

## Abstract

Stem cell self-renewal and the daughter cell differentiation are tightly regulated by the respective niches, which produce extrinsic cues to support the proper development. In *Drosophila* ovary, Dpp is secreted from germline stem cell (GSC) niche and activates the BMP signaling in GSCs for their self-renewal. Escort cells (ECs) in differentiation niche restrict Dpp outside the GSC niche and extend protrusions to help with proper differentiation of the GSC daughter cells. Here we provide evidence that loss of large Maf transcriptional factor Traffic jam (Tj) blocks GSC progeny differentiation. Spatio-temporal specific knockdown experiments indicate that Tj is required in pre-adult EC lineage for germline differentiation control. Further molecular and genetic analyses suggest that the defective germline differentiation caused by *tj*-depletion is partly attributed to the elevated *dpp* in the differentiation niche. Moreover, our study reveals that *tj*-depletion induces ectopic En expression outside the GSC niche, which contributes to the upregulated *dpp* expression in ECs as well as GSC progeny differentiation defect. Alternatively, loss of EC protrusions and decreased EC number elicited by *tj-*depletion may also partially contribute to the germline differentiation defect. Collectively, our findings suggest that Tj in ECs regulates germline differentiation by controlling the differentiation niche characteristics.

## Introduction

The *Drosophila* ovaries continually generate mature eggs in adulthood due to a stable population of self-renewable ovarian germline stem cells (GSCs). GSC progeny differentiation which ultimately generates a terminally differentiated egg is a stepwise process involving both cell-autonomous mechanisms and intercellular communications between GSC progeny and the surrounding ovarian somatic cells. The adult *Drosophila* females are born with a pair of ovaries, each of which contains 16–20 strings of ovarioles. The anterior-most structure of each ovariole is the germarium, which is followed by progressively maturing egg chambers^1^. At the tip of germarium, 2–3 GSCs are supported by GSC niche, including terminal filament (TF), cap cells (CpCs) and the most anterior escort cells (ECs)^2,3^. GSC divides asymmetrically to maintain self-renewal and produce a cystoblast (CB) simultaneously. Dpp and Gbb derived from GSC niche cells activate pMad (phosphorylated *Mother against daughter*) in GSCs, which transcriptionally represses *bam* (*Bag of marbles*)^4–6^. CB exits the GSC niche with attenuated BMP signaling activity, leading to the de-repression of *bam* transcription^4,5^. Then the CB undergoes an incomplete mitotic division, which produces a 2-cell cyst interconnected by the germline specific organelle called fusome^7^. Proper GSC progeny differentiation is also controlled extrinsically by a differentiation niche containing somatic ECs, which encapsulate GSC progeny with protrusions^8,9^. The physical interaction between ECs and germline is essential for germline differentiation^10–15^. Vice versa, differentiated GSC progeny might support the formation of EC protrusions^10^.

The EC-mediated molecular mechanisms to extrinsically modulate GSC progeny differentiation are under extensive study. Based on previous studies, suppression of Dally^16^ and expression of Tkv^17^ in ECs are necessary to restrict diffusion of GSC niche-derived Dpp within the range of one-cell-diameter. It has been shown that Dally suppression in ECs is modulated by MAPK signaling^18^ and Eggless^19^. Interestingly, Wnt signaling in ECs regulates the Tkv expression without repressing Dally and Dpp^17^. On the other hand, Lsd1^20,21^, Piwi^22,23^, COP9/Hh^24,25^ and H1^26^ have been reported to control GSC progeny differentiation through repressing Dpp expression in ECs. Moreover, Rho1^10^ and Bre1/Set1^27^ function in ECs to inhibit both Dpp and Dally production. Hh signaling antagonizes JAK/STAT activity to maintain an optimum Dpp expression^3^ and PRC1 represses the transcription of *dpp*-RB isoform in ECs^28^. In addition, Engrailed (En) has been shown to activate transcription of *dpp* in CpCs^29^. Besides all the above, the detailed molecular mechanisms for suppressing BMP ligands production in the differentiation niche still remain to be explored.

Traffic jam (Tj), also known as dMaf, is the only large Maf transcription factor in *Drosophila melanogaster*, whose homologs play pivotal roles in vertebrate development^30–34^. *tj* mRNA becomes detectable from late embryonic stage 12 onward in somatic gonadal precursor cells (SGPs)^33,35^. Tj protein has already been distributed in the nuclei of SGPs of embryonic gonads at stage 15–16^33^. And the expression is persisted in germline-associated somatic cells during ovarian development and oogenesis, including intermingled cells (ICs), CpCs, ECs and follicle cells (FCs)^33,36,37^. *tj* mutant ovaries are malformed and lack of ovariole subdivision^37,38^. Tj-mediated suppression of E-cadherin expression is required for SGPs to become ICs^39^. Moreover, Tj in CpCs inhibits the TF cell fate^37^. Considering Tj expression in ECs, we wonder if Tj plays essential extrinsic roles in controlling female germline differentiation.

Here we report evidence that Tj controls CB differentiation in a non-cell-autonomous manner during the pre-adult stage. *tj*-deficient pre-adult ECs produce ectopic En, which promotes *dpp* transcription and subsequently up-regulates BMP signaling activity in CBs, thereby causing differentiation defect. We further show that Tj is required in pre-adult ECs to maintain EC protrusions and establish a proper EC number. This study suggests new mechanisms by which Tj preserves the properties of differentiation niche to promote proper germline differentiation.

## Results

### Tj regulates germ cell differentiation in *Drosophila* ovaries

**Figure 1:**
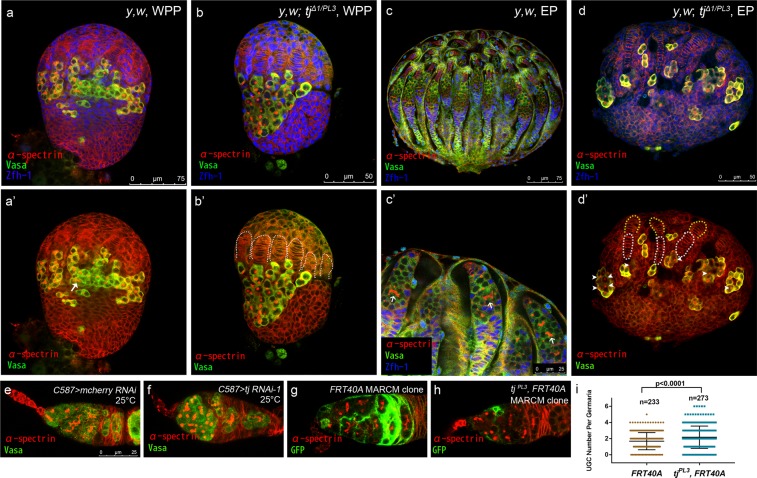
Tj is required for proper GSC progeny differentiation. **(a–d’)** Gonads are stained for α-spectrin (red), to mark spectrosomes and fusomes, Vasa (green), to mark germline, and Zfh-1 (blue), to mark somatic cells. **(a-b’)** White pre-pupal (WPP) gonads are examined. The control group **(a,a’)** contains branched fusomes, as indicated by white arrow. *tj* mutant gonad **(b,b’)** lacks branched fusome-containing-germline cyst. White dashed lines indicate TF stacks. **(c–d’)** Early pupal (EP) gonads are examined. The control group **(c,c’)** displays well-organized primordial ovarioles. White arrows indicate branched fusome-containing differentiating cysts. The *tj* mutant **(d,d’)** displays few differentiating germline cysts. White arrowheads indicate round fusome-containing germ cells. Yellow dash lines indicate TFs and white broken ovals indicate CpCs. **(e,f)** Germaria from newly eclosed flies (<1-day old) are stained for α-spectrin (red), to mark spectrosomes and fusomes, Vasa (green), to mark germline. The control group **(e)** shows normal number of CB. The *C587* > *tj RNAi-1* **(f)** group displays excess UGCs. **(g,h)** Mosaic germaria with MARCM clones are stained for α-spectrin (red), to mark spectrosomes and fusomes, GFP (green), to mark the clone cells positively. Germaria from newly eclosed flies (<1-day old) are examined. The control group (with *FRT40A* clone) **(g)** exhibits normal in germline differentiation. The *tj* mutant group (with *tj*^*PL3*^, *FRT40A* clone) **(h)** exhibits more spectrosome-containing cells. **(i)** Graph shows the UGC number in germaria of indicated genotypes. Error bars are shown as Means ± S.D. of each genotype. Scale bar for (**a–d’**) is shown in each panel. Scale bar for (**e–h**) is shown in panel (e). Anterior is up in (**a–d**) and left in (**e–h**).

It has been reported that loss of Tj blocks germ cell differentiation in *Drosophila* testis^33^. This prompted us to investigate a potential role of Tj in regulating female germline differentiation. We first assayed aberrant germ cell differentiation in *tj* mutant ovaries by immunofluorescence. Beginning at late third larval instar (LL3), primordial germ cells (PGCs) -derived germ cell differentiation initiates and proceeds in *Drosophila* female gonads^40,41^, producing differentiating germ cells including CBs and actively dividing cysts. Those germ cells at different developmental stages can be morphologically distinguished by the fusome, a germline specific cytoplasmic organelle which appears spherical in PGCs or GSCs and grows new branches after each cyst division^42^. At white pre-pupal stage (120 h AEL), germ cell differentiation in control gonads is evident by the presence of germline cysts containing branched fusomes, as visualized by staining with anti-α-spectrin antibody (4 out of 20 gonads examined, Fig. 1a,a’, white arrow). Compared with the control, differentiating germline cysts were detected at a smaller percentage in the gonads transheterozygous for *tj*^*Δ1*^ and *tj*^*PL3*^ (1 out of 20 gonads examined). The vast majority of germ cells in *tj*^*Δ1/PL3*^ gonads also harbored a spherical fusome (Fig. 1b,b’). Notably, PGCs formed a cluster and were surrounded by a layer of somatic cells labeled by anti-Zfh-1, as previously reported^33^. At early pupal stage (EP, 144 h AEL), the wild type gonads have developed into 18–20 primordial ovarioles. We checked each ovariole in the EP gonads and scored the frequency of ovarioles lacking branched fusomes. We found that all the ovarioles contained differentiating germline cysts in control gonads (180 primordial ovarioles, Fig. 1c,c’, white arrows). In contrast, about 80% primordial ovarioles of *tj* mutant EP gonads lacked germline cysts with branched fusomes, but exhibited spectrosome-containing germ cells accumulation (152 primordial ovarioles, Fig. 1d,d’, white arrowheads). CpCs were disc-shaped and arranged in a single row similar to TF cells (Fig. 1d’, white open ovals and yellow dashed lines). These results presented a similar phenotype to the observation of Panchal, *et al*.^37^. In addition, the ovaries from adult *tj*^*Δ1/PL3*^ were small and lacked mature germ cells, as previously reported^33,37^.

As Tj is expressed in IC/EC lineage in developing female gonads^33,36^, we sought to determine its extrinsic effects on germ cell differentiation in the ovaries. For this purpose, we knocked down the expression of *tj* specifically in somatic cells using either *UAS-tj RNAi-1* or *UAS-tj RNAi-2* driven by somatic specific *tj-GAL4*. Prior to the test, the RNAi knockdown (KD for short) efficiency was evaluated at the protein level using antibody against Tj in LL3 gonads. As previously reported, Tj protein is concentrated in ICs since L3^33,36^ (Fig. S1a,a’). Our results revealed that these two independent RNAi lines driven by *tj-GAL4* reduced the Tj protein levels substantially in somatic lineage (Fig. S1b–c’) and led to a drastic phenotype similar to *tj* transhterozygotes (Fig. S1d–f’). However, we could only get severely degenerated ovaries after eclosion probably due to a lack of mature germline or a dramatic loss of ECs. To restrict the expression time window for *tj* RNAi, *tj-GAL4* combined with *tub-GAL80*^*ts*^ was used. The flies were initially raised at 18 °C and then switched to 29 °C at different time points during pupal stage or adulthood. However, once the temperature shift carried out, the ovaries from any experimental groups degenerated rapidly, even though the flies were switched to 25 °C. This technical setback prompted us to express *tj* RNAi with *C587-GAL4*, another somatic specific driver active in developing gonad and adult germarium^6,43^. This led to a minor impairment of Tj expression since larval stage (Fig. S2a–i”), thus bypassing the strong phenotype of *tj* loss-of-function in earlier stage. In control germaria (Fig. 1e), 0 to 4 CBs were identified by spherical fusomes located not immediately adjacent to CpCs. As shown in Fig. 1f, reduced Tj expression in the somatic lineage led to a remarkable increase of spherical fusome-containing single germ cells classified as undifferentiated germ cells (UGCs). Note that the interaction between PGCs and ICs was not affected by *tj* KD in the LL3 gonads (Fig. S2a–c”), excluding the possibility that PGC cluster contributed to the adult phenotype. Next, we generated MARCM^44^ (the Mosaic Analysis with a Repressible Cell Marker) clones homozygous for *tj*^*PL3*^ to further analyze the phenotype in adulthood. The control (*FRT40A*) and the *tj* mutant (*FRT40A, tj*^*PL3*^) clones were induced in parallel (see Methods). The ovaries were stained for GFP as a positive clone marker and for α-spectrin to label the fusome. In control germaria (Fig. 1g), most of ECs were labeled with GFP. Control EC clones almost never exhibited UGC accumulation, with only one exception that contained 5 UGCs (1 out of 233 germaria). Although the percentage of germaria with clonal EC was similar to the control group, only small clones of ECs mutant for *tj* were recovered in this condition (Fig. 1h). There was 6.23% of the germaria (17 out of 273 germaria with clonal EC and without clonal TF and CpCs) exhibiting excess UGCs (Fig. 1i). Lower penetrance is potentially owing to the smaller clone size. Taken together, these data indicate that Tj is required to regulate GSC progeny differentiation in *Drosophila* ovarian development.

### Tj functions mainly in pre-adult ECs to control proper germ cell differentiation

**Figure 2:**
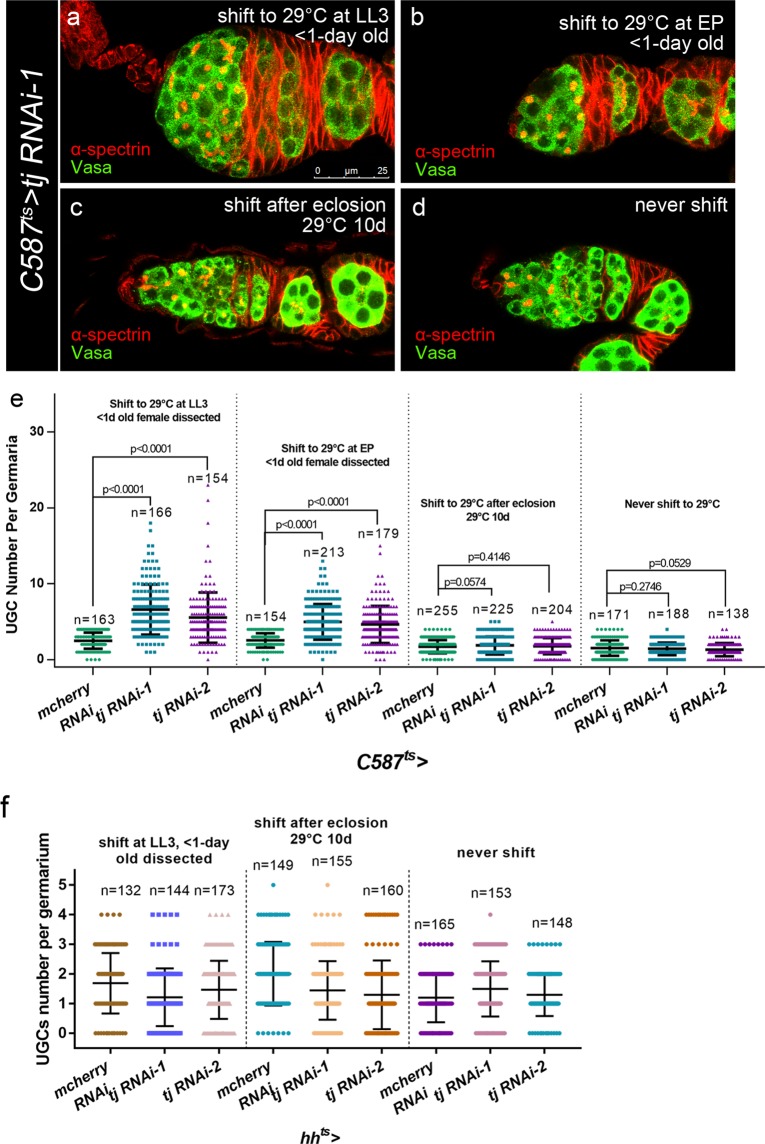
GSC progeny differentiation defects due to *tj* KD predominantly arise in EC lineage during pre-adult stage. **(a–d)** Germaria from *C587*^*ts*^ > *tj RNAi-1* flies are stained for α-spectrin (red), to mark spectrosomes and fusomes, Vasa (green), to mark germline. *C587*^*ts*^-driven other *tj* RNAi lines present similar phenotype. **(a,b)** Germaria from newly eclosed flies (<1-day old), which are raised at 18 °C up to LL3 **(a)** or EP stage **(b**) and then maintained at 29 °C till eclosion, display UGC accumulation. **(c,d**) Germaria from 10-day-old females, which are raised at 18 °C up to eclosion and then shifted to 29 °C **(c)** or still maintained at 18 °C (**d)**, present normal procedure of germline differentiation. (**e,f**) Graph shows the UGC number in germaria of indicated genotypes. Error bars are shown as Means ± S.D. of each genotype. Scale bar is shown in panel (a). Anterior is always to the left.

To characterize the developmental stage-specific requirements for Tj in GSC progeny differentiation control, *C587-GAL4* combined with *tub-GAL80*^*ts*^ (*C587*^*ts*^ for short) was employed to control *tj* RNAi expression at a particular stage. Available *UAS-tj* RNAi transgenic lines and control *UAS-mcherry RNAi* were crossed to *C587*^*ts*^ and the progeny were subjected to time-course analysis. Flies were maintained at the permissive temperature (18 °C) until a given developmental stage and then shifted to the restrictive temperature (29 °C) for several days in order to knock down *tj* specifically during developmental or adult stage. First, the newly eclosed females (<1-day old) with developmental knockdown of *tj* were examined. Shifting the animals of *tj* KD to 29 °C at either LL3 (Fig. 2a) or EP (Fig. 2b) stage resulted in a striking expansion of UGC number. We subsequently examined the 10-day old females shifted to 29 °C immediately after eclosion (Fig. 2c). However, we did not find UGC phenotype with statistical significance upon adult-specific depletion of *tj*, in which only about 1% germaria exhibited 5 UGCs. As a control group, animals were raised at 18 °C until eclosion, and then kept at 18 °C for 10 days. Ovaries from these females exhibited a normal number of CB (Fig. 2d). These data demonstrate that Tj is critically required during developmental stage, rather than adulthood, for proper germline differentiation (Figs 2e and S3a).

It is known that *C587-GAL4* is active in most of the somatic lineage in developing female gonads^6,43^, which will differentiate into more specialized cell types in germaria during subsequent development, such as TF, CpC, IC/EC and early FCs^45^. To determine Tj functions in which cell types, *hh-GAL4* combined with *tub-GAL80*^*ts*^ (*hh*^*ts*^ for short) was used to express *tj* RNAi exclusively in TF and CpCs since their specification^39^. Flies were initially kept at 18 °C and shifted to 29 °C since LL3. Interestingly, the *tj* KD germaria from newly eclosed females (<1-day old) displayed greatly diminished Tj antibody staining in CpCs and normal germ cell differentiation (Figs 2f and S3b–g). However, about 50% of the germaria still exhibited detectable expression of Tj in 1–2 CpCs. Thus, we could not exclude the possibility that the remaining amount of Tj is enough to maintain normal germline differentiation. Taken together with the observation that germaria bearing *tj* mutant EC clones presented UGCs accumulation, we argue that Tj is predominantly required in pre-adult ECs for regulating *Drosophila* ovarian germ cell differentiation.

### Tj is required in pre-adult ECs to promote CB differentiation by restricting BMP signaling

**Figure 3:**
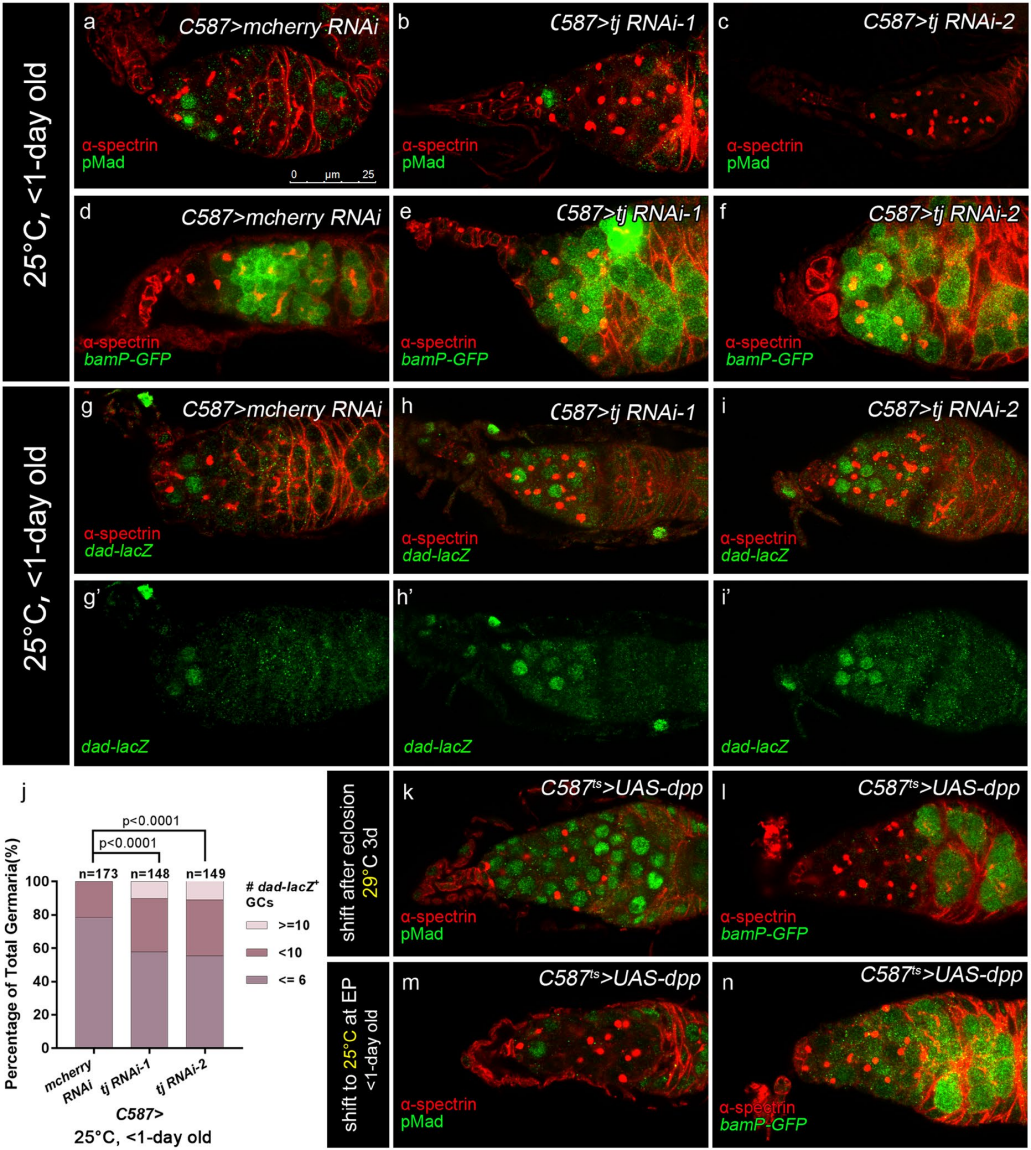
Loss of Tj in ECs results in expanded BMP signaling. **(a–c,k** & **m)** Germaria are stained for pMad (green), to indicate the BMP pathway activity, α-spectrin (red), to mark spectrosomes and fusomes. In germaria from newly eclosed flies (<1-day old), the control group **(a)** displays dense pMad staining in GSCs. The *C587* > *tj RNAi-1* **(b)** and *C587* > *tj RNAi-2* **(c)** groups display excess UGCs negative for pMad. **(d–f, l & n)** Germaria are stained for α-spectrin (red) to mark spectrosomes and fusomes. The *bamP-GFP/+* (green) is introduced to indicate the transcriptional activity of *bam*. In germaria from newly eclosed flies (<1-day old), the control group **(d)** displays the absence of *bamP-GFP* in GSCs but upregulation in CBs. The *C587* > *tj RNAi-1* **(e)** and *C587* > *tj RNAi-2* **(f)** groups display *bamP-GFP-*positive UGCs. **(g–i’)** Germaria from newly eclosed flies (<1-day old) are stained for α-spectrin (red), to mark spectrosomes and fusomes. The *dad-lacZ*/+ is introduced to indicate BMP pathway activity (marked by β-gal, green). The control group **(g,g’)** presents high level of *dad-lacZ* activity in GSCs and intermediate CBs. The *C587* > *tj RNAi-1* **(h,h’)** and *C587* > *tj RNAi-2* **(i,i’)** groups present *dad-lacZ*-positive UGCs outside the GSC niche. **(j)** Graph shows the percentage of germaria that contain the indicated number of *dad-lacZ*-positive germ cells for each genotype. **(k,l)** *C587*^*ts*^ > *UAS-dpp* flies are maintained at 18 °C up to eclosion and then shifted to 29 °C for 2–3 days before dissection. The germaria are full of pMad-positive UGCs **(k)** and *bamP-GFP-*negative UGCs **(l)**. **(m,n)** Germaria from newly eclosed flies (<1-day old) of *C587*^*ts*^ > *UAS-dpp*, which are maintained at 18 °C up to EP stage and then maintained at 25 °C till eclosion, exhibit excess pMad-negative UGCs **(m)** and *bamP-GFP*-positive UGCs **(n)**. Scale bar is shown in panel (a). Anterior is always to the left.

Previous findings suggest that ectopic BMP signaling activated in germline differentiation zone results in an expansion of GSC/CB-like cells^10,19–27,46^. To determine if Tj is required extrinsically for restricting the BMP signaling activity outside the GSC niche, we examined the BMP signaling activity by various reporters in flies expressing *tj* RNAi by *C587-GAL4*. In these experiments, crosses were performed and the resulting progeny were raised at 25 °C, and newly eclosed females (<1-day old) were dissected for detection. We first evaluated BMP signaling activity with antibody against phosphorylated Mad (pMad). In wild type germaria, pMad expression is restricted to the GSCs (Fig. 3a). Unexpectedly, in *tj* KD germaria, the pMad was barely detectable in UGCs (Fig. 3b,c). GFP reporter labeling the activity of the *bam* transcription, *bamP-GFP*^4,5^, was highly visible in all UGCs in *tj* KD germaria (Fig. 3e,f). Whereas it is normally absent in GSCs and first become detectable in CBs and upregulated in mitotic cysts (Fig. 3d). Next, we checked the enhancer trap *dad-lacZ*^47^ which had been reported as a low threshold reporter of BMP signaling^3^. *dad* (*daughters against dpp)* is a target gene for BMP signaling and the expression of *dad-lacZ* in germline is confined to GSCs and intermediate CBs in control germaria (Fig. 3g,g’). In contrast, the number of *dad-lacZ*-positive germ cell was significantly expanded in germaria of *C587* > *tj RNAi* females (Fig. 3h–j). These results indicate that *tj* depletion in pre-adult ECs causes an up-regulation of BMP signaling outside GSC niche. Moreover, the UGCs are blocked at a late CB stage.

To gain more evidence to support our observation, we further overexpressed *dpp* with *C587*^*ts*^, which has been demonstrated to result in the formation of the accumulated GSC-like cells^10,48^. First the crosses were set up and the resulting progeny were raised at 18 °C until eclosion and then kept at 29 °C for 2–3 days. As expected, the accumulated UGCs in germaria exhibited robust pMad staining (Fig. 3k) and were devoid of *bamP-GFP* expression (Fig. 3l). As a second strategy for overexpression, the crosses were set up and the resulting progeny were raised at 18 °C until EP stage. And then the flies were shifted to 25 °C for a moderate overexpression of *dpp* in pre-adult ECs, as *tub-GAL80*^*ts*^ is not fully functional at this temperature^49^. The newly born ovaries of those females exhibited an expansion of UGCs devoid of pMad staining (Fig. 3m) and positive for *bamP-GFP* (Fig. 3n). These results indicate that a moderate elevation of *dpp* in pre-adult ECs results in an accumulation of CB-like germ cells. Taken together, we argue that depletion of *tj* in pre-adult ECs may induce an elevation of *dpp* outside the GSC niche.

### Tj is required extrinsically to control germline differentiation by repressing *dpp* expression

**Figure 4:**
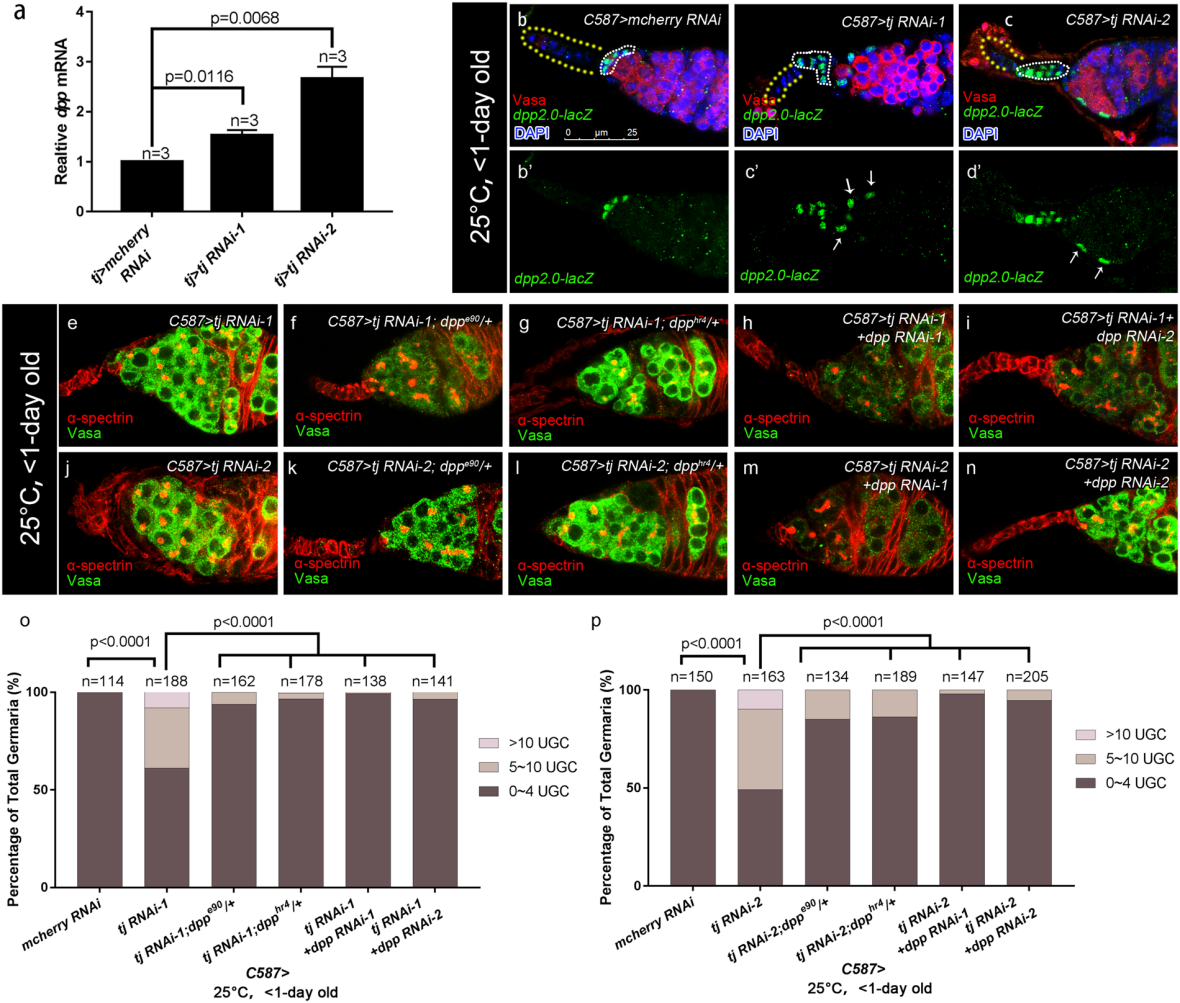
Ectopic *dpp* partly contributes to the germline differentiation defect in *C587*-mediated *tj* KD germaria. **(a)** Result of qRT-PCR shows that the *dpp* mRNA is increased by 1.53 ± 0.06 fold in *tj* > *tj RNAi-1* ovaries and 2.66 ± 0.14 fold in that of *tj* > *tj RNAi-2*. Data are shown as Means ± S.D. of three independent experiments. (**b–d’)** Germaria from newly eclosed flies (<1-day old) are stained for Vasa (red), to mark germline. The *dpp2.0-lacZ*/+ is introduced to indicate *dpp* transcription activity (marked by β-gal, green). DAPI is blue. Yellow dashed lines indicate TFs. White broken ovals indicate CpC clusters. The control group **(b,b’)** exhibits β-gal staining only in cap cells. The *C587* > *tj RNAi-1* **(c,c’)** and *C587* > *tj RNAi-2* **(d,d’)** groups show ectopic *dpp2.0-lacZ* activity in some ECs, as indicated by white arrows. **(e–n)** Germaria from newly eclosed flies (<1-day old) are stained for α-spectrin (red), to mark spectrosomes and fusomes, Vasa (green) to mark germline. The *C587* > *tj RNAi-1* group **(e)** exhibits excess UGCs. The *C587* > *tj RNAi-1; dppe90*/+ **(f)**, *C587* > *tj RNAi-1; dpp*^*hr4*^/+ **(g)**, *C587* > *tj RNAi-1* + *dpp RNAi-1* **(h)** and *C587* > *tj RNAi-1* + *dpp RNAi-2* **(i)** groups exhibit reduced UGC accumulation. **(j–n)** The *C587* > *tj RNAi-2* **(j)** group exhibits excess UGCs. The *C587* > *tj RNAi-2; dpp*^*e90*^/+ **(k)**, *C587* > *tj RNAi-2; dpp*^*hr4*^/+ **(l)**, *C587* > *tj RNAi-2* + *dpp RNAi-1* **(m)** and *C587* > *tj RNAi-2* + *dpp RNAi-2* **(n)** groups exhibit reduced UGC accumulation. **(o,p)** Graphs show the percentage of germaria that contain the indicated number of UGC for each genotype. Scale bar is shown in panel (b). Anterior is always to the left.

To further elucidate mechanisms underlying expanded BMP signaling elicited by *tj* KD, quantitative RT-PCR was conducted to determine the *dpp* expression at the transcriptional level. Due to the limitation of the technique, we were not able to purify the ECs from ovaries. We isolated ovary RNAs of virgins carrying *tj-GAL4* > *tj RNAi* or *tj-GAL4* > *mcherry RNAi*, as the ovaries of each genotype were morphologically similar and contain few egg chambers, which made the comparison more valid. Notably, *dpp* mRNA was elevated in *tj-GAL4*-mediated *tj* RNAi ovaries compared with the control group (Fig. 4a), suggesting that Tj mediates the repression of *dpp* transcription. To visualize the changes of *dpp* expression in germaria, two recently published enhancer trap lines, *dpp2.0-lacZ*^29^ and *P4-lacZ*^28^, were employed to trace the *dpp* transcription *in vivo*. Both reporters were evaluated when expressing *tj* RNAi using *C587*-*GAL4* at 25 °C. In the wild type germaria, expression of *dpp2.0-lacZ*, which faithfully recapitulates *dpp* expression in germarium^29^, is strongly visible in CpCs (Fig. 4b,b’, open ovals) and occasionally found in 1–3 ECs (about 12% of total control germaria, n = 640, Fig. S4a,a’, white arrowhead) as reported. Remarkably, *C587-GAL4* driven expression of *tj* RNAi caused a more frequent *dpp2.0-lacZ* expression in ECs (Fig. 4c–d’, white arrows). On the other hand, *tj*-depleted ECs also showed an ectopic *P4-lacZ* activity, which is typically expressed in TF and CpCs in wild type germaria (Fig. S4b–e). Here, ECs could be distinguishable from other somatic cells by their position and nuclear morphology. CpCs were identified with round- or disc-shaped nuclei upon DAPI staining. ECs were identified with triangle-shaped or flattened and curved spindle-shaped nuclei upon DAPI staining. It is noteworthy that CpCs adopted a stalk-like or branched organization (Figs 4b–d and S4b–d, white broken ovals and yellow dashed lines) and GSC number decreased (Fig. S4f,g) in gemaria expressing *tj* RNAi by *C587-GAL4*, which are similar to previous findings^37^. Thus, the ectopic *dpp-lacZ*-expressing cells might not be a mislocalization of CpCs. Meanwhile, TFC and CpC number were not affected by *C587*-mediated *tj* RNAi, as manifested by *Dl-lacZ* (Fig. S5a–b’) and *hh-lacZ* respectively (Fig. S5c–d’). Furthermore, *C587* > *Flp*-mediated generation of CpC clones during niche formation resulted in similar clone size of CpCs between control and *tj*^*PL3*^ mutant mosaic germaria (Fig. S5e–j”’). These results implicate that *tj*-depletion does not induce expansion of TFCs or CpCs, and the ectopic *dpp-lacZ*-expressing cells are ECs that display a characteristic feature of CpCs.

Then we tested whether excess Dpp signaling outside the GSC niche is responsible for accumulated UGC in *tj* KD germaria. To this aim, the UGC number was assessed in germaria of reducing *dpp* expression levels in flies expressing *tj* RNAi by *C587-GAL4*. Heterozygotes of either *dpp* alleles (*dpp*^*hr4*^ or *dpp*^*e90*^) in the presence of *tj* KD exhibited a partial suppression of UGC accumulation. Likewise, *C587*-mediated double knockdown of *dpp* and *tj* led to a significant reduction of the UGC number (Fig. 4e–p). Furthermore, similar results were obtained with a pupal-specific *tj dpp* double knockdown with *C587*^*ts*^ (Fig. S6a,b). Taken together, our results support the idea that Tj in pre-adult ECs regulates CB differentiation at least partly by restricting *dpp* expression. The incomplete rescue could be attributed to the incomplete knockdown of *dpp* or an unidentified mechanism independent of *dpp* repression.

### En dysregulation in ECs with *tj* depletion contributes to the *dpp* expansion in germaria

**Figure 5:**
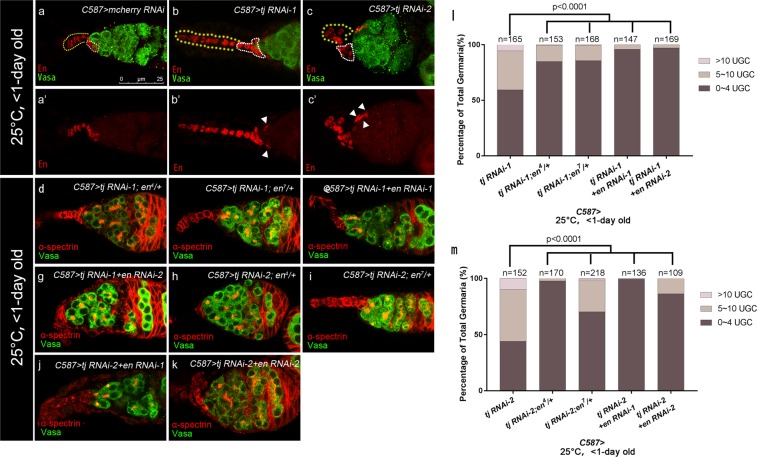
Engrailed misregulation partly contributes to the germline differentiation defect in *tj* KD ECs. (**a–c’**) Germaria from newly eclosed flies (<1-day old) are stained for Vasa (green), to mark germline, En (red), to mark TF and CpCs. Yellow dashed lines indicate TFs and white broken ovals indicate CpC clusters. The control group **(a**,**a’)** displays specific En expression in TF and CpCs. The *C587* > *tj RNAi-1* **(b,b’)** and *C587* > *tj RNAi-2* **(c,c’)** group show ectopic En staining in some ECs, as indicated by white arrowheads. **(d**–**k)** Germaria from newly eclosed flies (<1-day old) are stained for α-spectrin (red), to mark spectrosomes and fusomes, Vasa (green), to mark germline. The *C587* > *tj RNAi-1; en*^4^/+ **(d)**, *C587* > *tj RNAi-1; en*^7^/+ **(e)**, *C587* > *tj RNAi-1* + *en RNAi-1* **(f)** and *C587* > *tj RNAi-1* + *en RNAi-2* **(g)** groups exhibit a reduced UGCs accumulation, compared with Fig. 4e. The *C587* > *tj RNAi-2; en*^4^/+ **(h)**, *C587* > *tj RNAi-2; en*^7^/+ **(i)**, *C587* > *tj RNAi-2* + *en RNAi-1* **(j)** and *C587* > *tj RNAi-2* + *en RNAi-2* **(k)** groups exhibit a reduced UGCs accumulation, compared with Fig. 4j. (**l**, **m)** Graphs show the percentage of germaria that contain the indicated number of UGC for each genotype. Scale bar is shown in panel (a). Anterior is always to the left.

One recent study confirmed that Engrailed (En), a homeodomain-containing transcription factor, regulates *dpp* expression in CpCs by binding to a 2.0 kb fragment 5′ enhancer region of *dpp*^29^. And forced expression of En in ECs causes UGC accumulation as well as ectopic expression of *dpp2.0-lacZ*^21,29^. These results raise a possibility that the ectopic En could induce UGCs accumulation in *tj* KD germaria through regulating *dpp* transcription. To test this hypothesis, we investigated whether reduced expression of *tj* causes an aberrant change in En expression in germaria. The crosses were performed and the resulting progeny were raised at 25 °C, and newly eclosed females (<1-day old) were dissected for detection. Immunostaining revealed that En is specifically expressed in the nuclei of TF and CpCs in control germaria^21,50,51^ (Fig. 5a,a’). In contrast, some ECs in the anterior portion of the germaria exhibited ectopic En expression when *tj* was knocked down (Fig. 5b–c’, white arrowheads). As expected, we also found the deformed TF and CpC clusters similar to that have been previously reported^37^ (Fig. 5b–c’, white broken ovals and yellow dashed lines). And ECs were distinguishable from other somatic cells by their position and nuclear morphology, as stated above. Similarly, ectopic En expression in ECs was caused by pupal-specific, but not adult-specific *tj* KD (Fig. S7a–c’, white arrows and data not shown). To link the ectopic En expression with the UGC accumulation elicited by *tj* KD, the effect of heterozygous *en* allele or *UAS*-*en* RNAi lines on the *tj* RNAi differentiation phenotype was examined. Phonotypical analysis showed that compromised *en* expression significantly suppressed germline differentiation defects induced by *tj* RNAi (Fig. 5d–m). Similarly, pupal-specific *tj en* double knockdown with *C587*^*ts*^ significantly repressed the *tj* KD UGC phenotype (Fig. S6a,b). These results indicate that Tj acts in pre-adult ECs for germ cell differentiation control partially through negative regulation of En.

**Figure 6:**
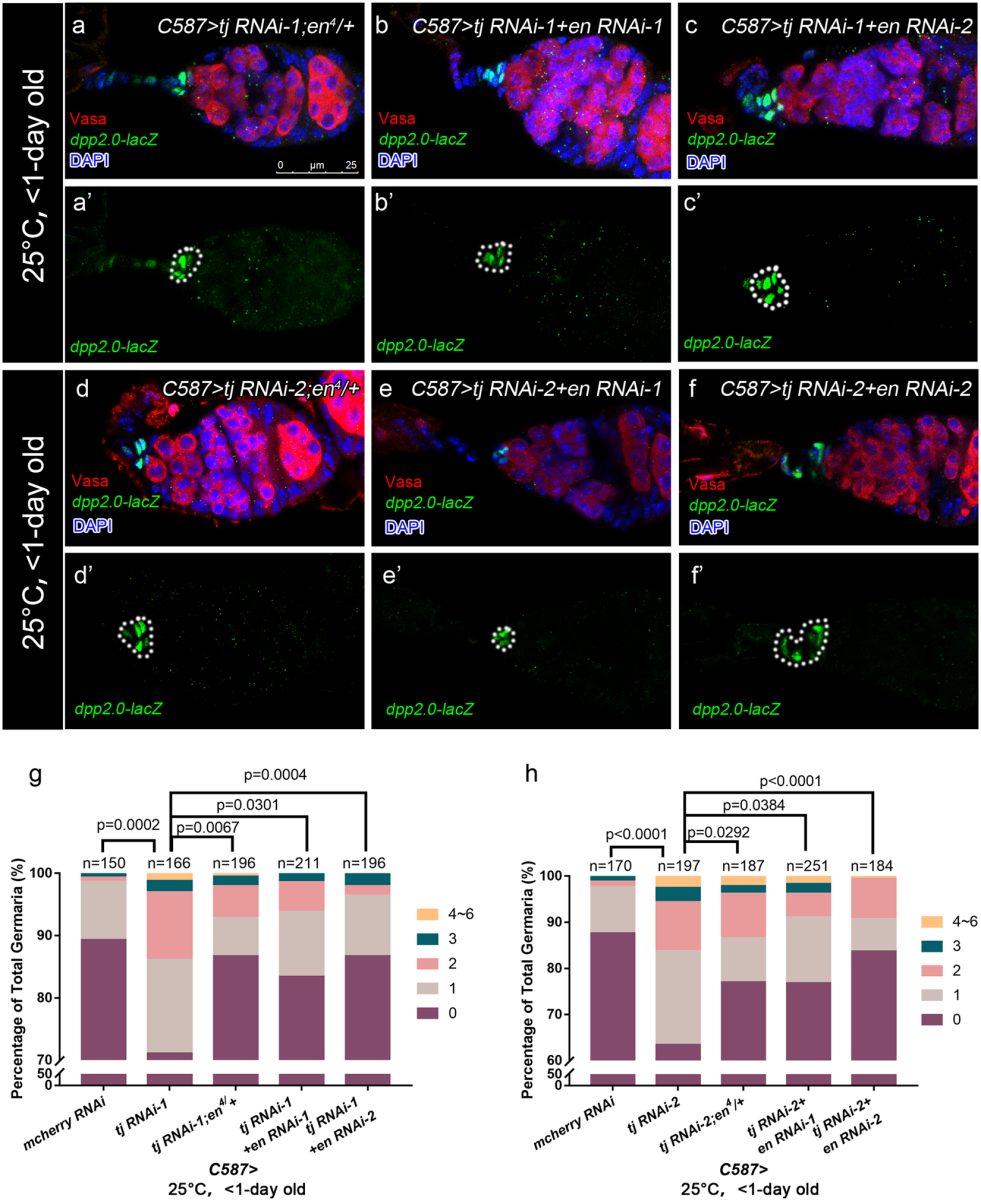
Tj represses *dpp* expression in ECs partially through En. **(a–f’**) Germaria from newly eclosed flies (<1-day old) are stained for Vasa (red) to mark germline. The *dpp2.0-lacZ*/+ is introduced to indicate *dpp* transcription activity (marked by β-gal, green). DAPI is blue. White broken ovals indicate CpC clusters. The *C587* > *tj RNAi-1; en*^4^/+ **(a,a’)**, *C587* > *tj RNAi-1* + *en RNAi-1* **(b,b’)** and *C587* > *tj RNAi-1* + *en RNAi-2* **(c,c’)** groups exhibit relieved expression of *dpp2.0-lacZ* in ECs, compared with Fig. 4(c,c’). The *C587* > *tj RNAi-2; en*^4^/+ **(d,d’)**, *C587* > *tj RNAi-2* + *en RNAi-1* **(e,e’)** and *C587* > *tj RNAi-2* + *en RNAi-2* **(f,f’)** groups exhibit relieved expression of *dpp2.0-lacZ* in ECs, compared with Fig. 4(d,d’). **(g,h)** Graphs show the percentage of germaria that contain a given number of EC with ectopic *dpp2.0-lacZ* expression of indicated genotypes.

**Figure 7:**
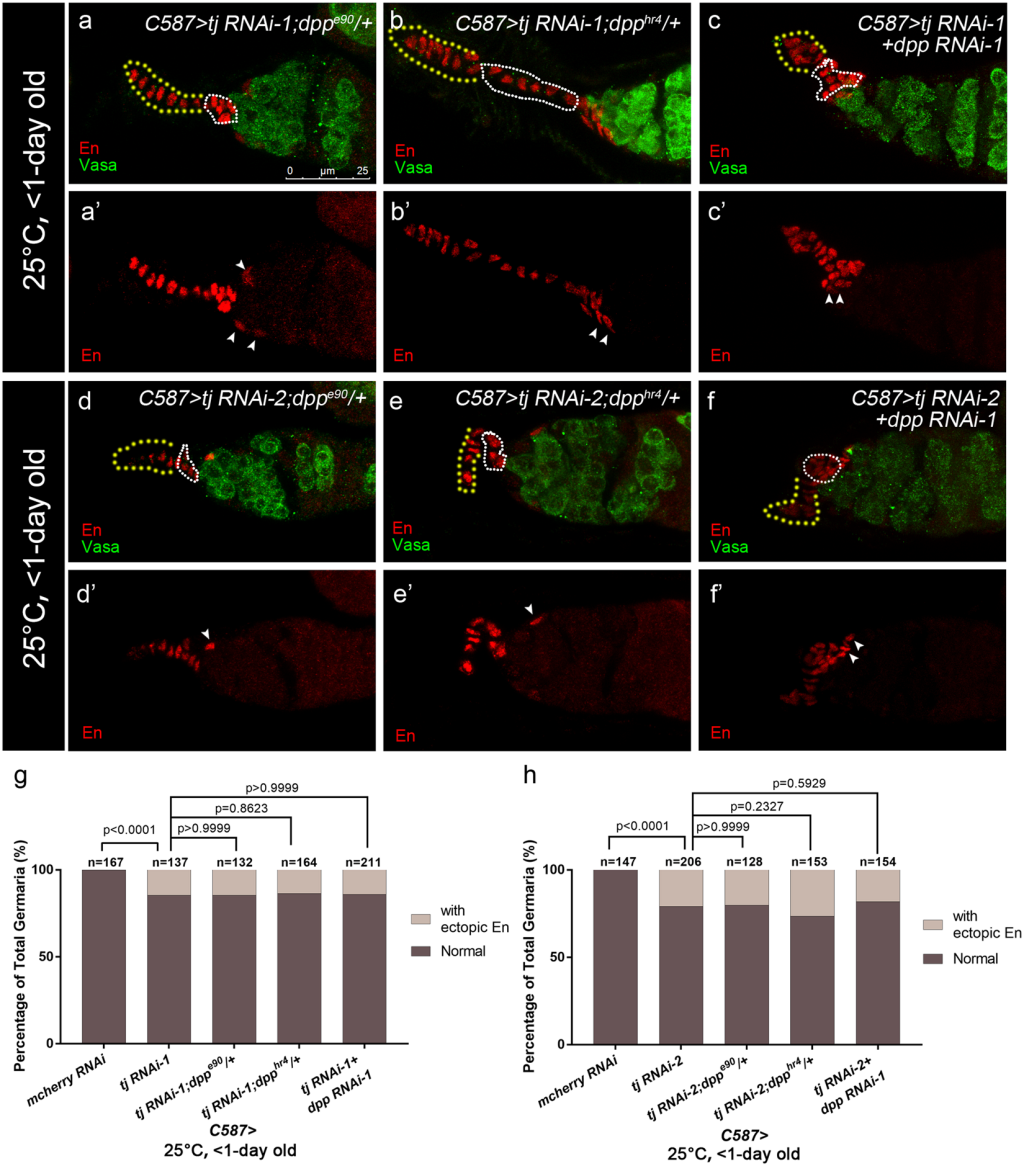
En acts upstream of Dpp. **(a–f’)** Germaria from newly eclosed flies (<1-day old) are stained for Vasa (green), to mark germline, En (red) to mark TF and CpCs. Yellow dashed lines indicate TFs and white broken ovals indicate CpC clusters. The *C587* > *tj RNAi-1; dpp*^*e90*^/+ **(a,a’)**, *C587* > *tj RNAi-1; dpp*^*hr4*^/+ **(b,b’)**, *C587* > *tj RNAi-1* + *dpp RNAi-1* **(c,c’)***, C587* > *tj RNAi-2; dpp*^*e90*^/+ **(d,d’)**, *C587* > *tj RNAi-2; dpp*^*hr4*^/+ **(e,e’)** and *C587* > *tj RNAi-2* + *dpp RNAi-1* **(f,f’)** groups exhibit ectopic En expression in ECs, as indicated by white arrowheads. **(g,h)** Graphs show the percentage of germaria containing ectopic En expression in ECs of indicated genotypes. Scale bar is shown in panel (a). Anterior is always to the left.

Given that En regulates *dpp* transcription in the GSC niche, we reasoned that ectopic En expression induced by *tj* KD might upregulate *dpp* transcription. To verify this assumption, we tested whether attenuation of *en* expression in *tj* KD germaria reduces the ectopic *dpp* expression. As expected, heterozygous *en* allele or *en* RNAi partially suppressed elevated *dpp* expression in *tj* KD germaria, as indicated by a significant decline in the number of EC positive for *dpp2.0-lacZ* (Fig. 6a–h). On the contrary, the ectopic En staining in *tj* KD ECs remained statistically unchanged when heterozygous *dpp* allele or *UAS*-*dpp* RNAi lines were present (Fig. 7a–h, white arrow heads). These results support the idea that Tj functions in ECs to control GSC progeny differentiation partly by En-regulated restriction of *dpp* expression.

### Tj regulates EC protrusions cell-autonomously in an En-independent context

**Figure 8:**
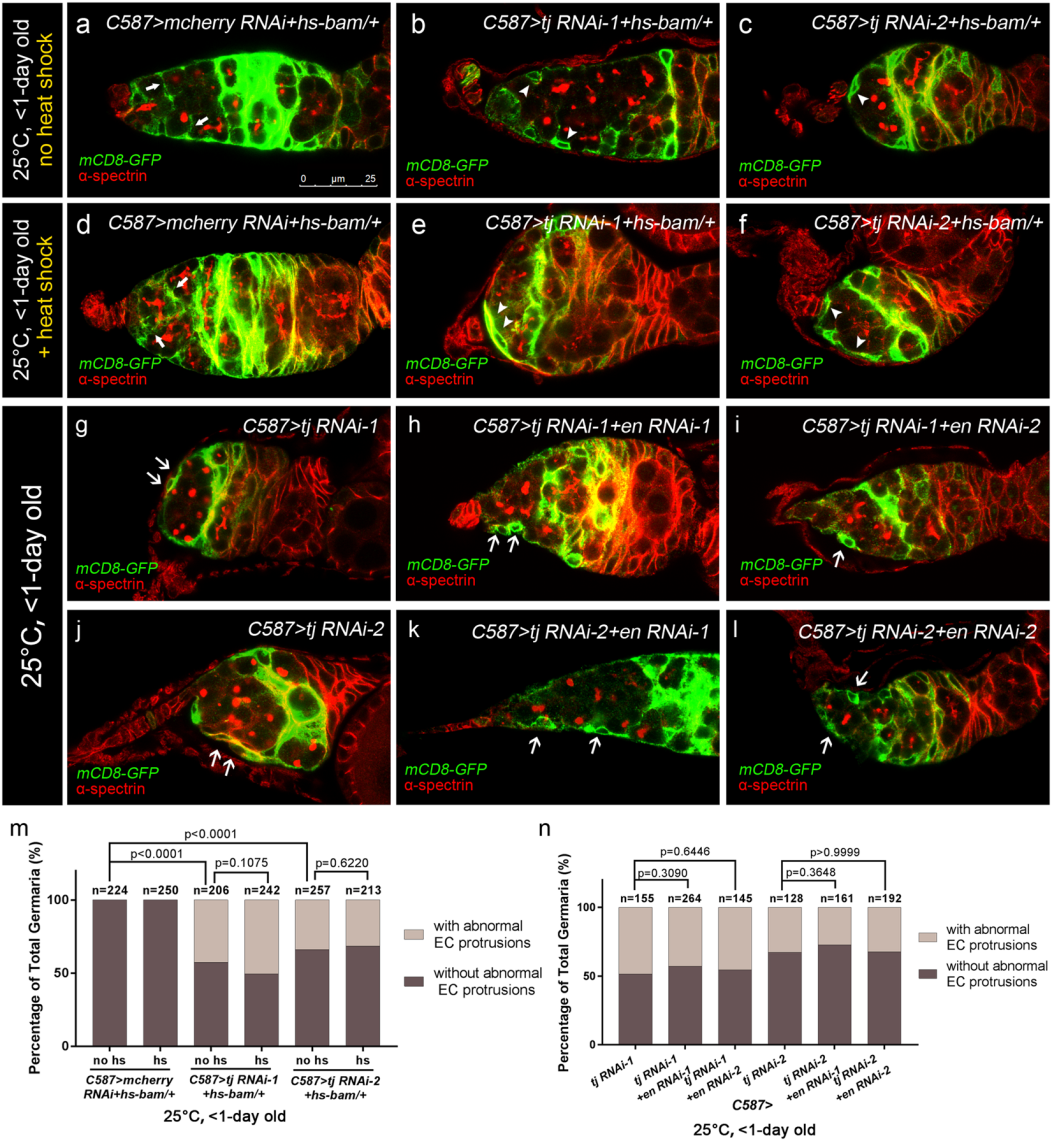
Tj controls EC protrusions independent of En. **(a–l)** Germaria from newly born (<1-day old) females are stained for α-spectrin (red) to mark spectrosomes and fusomes. *C587-*driven *UAS-mCD8-GFP* (green) visualizes the EC protrusions. **(a–f)** The *hs-bam*/+ is introduced. (**a–c**) Ovaries from females without heat shock treatment are examined. The control group **(a)** displays normal protrusions extended by ECs. White arrows indicate the EC protrusions separating developing germline cysts. The *C587* > *tj RNAi-1* + *hs-bam*/+ **(b)** and *C587* > *tj RNAi-2* + *hs-bam*/+ **(c)** groups exhibit abnormal EC morphology as well as UGCs accumulation. White arrowheads indicate the absence of EC protrusions. **(d–f)** Ovaries from females with heat shock treatment during pupal stage are examined. The control group **(d)** exhibits branched fusome-containing germline closed to niche cells. White arrows indicate the protrusions extended by ECs. The *C587* > *tj RNAi-1* + *hs-bam*/+ **(e)** or *C587* > *tj RNAi-2* + *hs-bam*/+ **(f)** groups exhibit no UGCs in the germaria. White arrowheads indicate the absence of EC protrusions. The *C587* > *tj RNAi-1* **(g)**, *C587* > *tj RNAi-1* + *en RNAi-1* **(h)**, *C587* > *tj RNAi-1* + *en RNAi-2* **(i)**, *C587* > *tj RNAi-2* **(j)**, *C587* > *tj RNAi-2* + *en RNAi-1* **(k)** and *C587* > *tj RNAi-2* + *en RNAi-2* **(l)** groups display abnormal EC protrusions. White arrows indicate the absence of EC protrusions. **(m,n)** The graph shows the percentage of germaria that contain EC with abnormal protrusions of indicated genotypes. Scale bar is showed in panel (a). Anterior is always to the left.

Abundant studies have demonstrated that EC protrusions-mediated interactions with germ cells are important for proper germline differentiation^10,12,21,23–25,46^. Given that Tj participates in the interaction between ICs and PGCs^33^, we further determined if *C587*-mediated *tj* depletion impairs EC protrusions. To test this, *UAS-mCD8-GFP* combined with *C587-GAL4* was used to visualize the EC protrusions. The crosses were set up and the progeny were maintained at 25 °C and newly eclosed females (<1-day old) were dissected for detection. In control germaria, EC protrusions were detected to encapsulate the germ cells outside the GSC niche (Fig. 8a, white arrows). Interestingly, some of the ECs lost protrusions and left germ cells unwrapped in *tj* KD flies (Fig. 8b,c, white arrowheads). Similar results were obtained for pupal-specific *tj* KD with *C587*^*ts*^. As visualized by anti-Coracle staining, ECs in germaria from newly eclosed females failed to penetrate into the germ cells (Fig. S8a–c’). However, no evidence of significant impairment in EC protrusions was found in *C587*^*ts*^-mediated adult-specific *tj* KD germaria. As the vast majority (about 99%) of the germaria exhibited normal EC protrusions (Fig. S8d–f’). These results are consistent with Tj mainly functioning in the pre-adult stage to prevent the formation of supernumerary UGCs. In addition, the MARCM analysis displayed small protrusions of *tj* clonal ECs (Fig. S8g–i, white arrows), which is consistent with the results from previous studies that wild type neighbors stabilized mutant EC extensions to encapsulate germ cells^15^. Thus, we conclude that Tj is required for proper EC protrusions, helping to facilitate germline differentiation.

To further support our conclusion, we took an advantage of *hs-bam* transgene, in which *bam* expression is under the control of the heat-shock inducible promoter, to induce germline differentiation in *tj* KD germaria. The heat shock was conducted at the pupal stage (see Methods) and the newly eclosed females (<1-day old) were dissected for detection. Heat-shock induced GSC differentiation resulted in differentiating cysts, instead of GSCs, occupied the tip of germaria in control groups, whereas the EC protrusions remained extended and wrapped the germ cells underneath (Fig. 8d, white arrows). Similarly, *C587* > *tj RNAi* germaria were occupied with multiple cell-cysts under heat-shock induced *bam* expression. However, no restoration of EC protrusions was observed (Fig. 8e,f, white arrowheads). In short, forced differentiation of UGC was not able to suppress the EC morphology defect induced by *tj-*depletion (Fig. 8m). Altogether, we conclude a cell-autonomous contribution of Tj in regulating EC protrusions.

To test whether Tj regulates EC protrusions through En, *en* RNAi and *tj* RNAi were coexpressed using *C587-GAL4* at 25 °C. No restoration of EC morphology defects was observed following co-expression of *en* RNAi in newly eclosed (<1-day old) females, suggesting that En is dispensable for *tj*-mediated regulation of EC protrusions (Fig. 8g–l,n).

### Tj regulates the establishment of a proper EC number in an En-independent context

**Figure 9:**
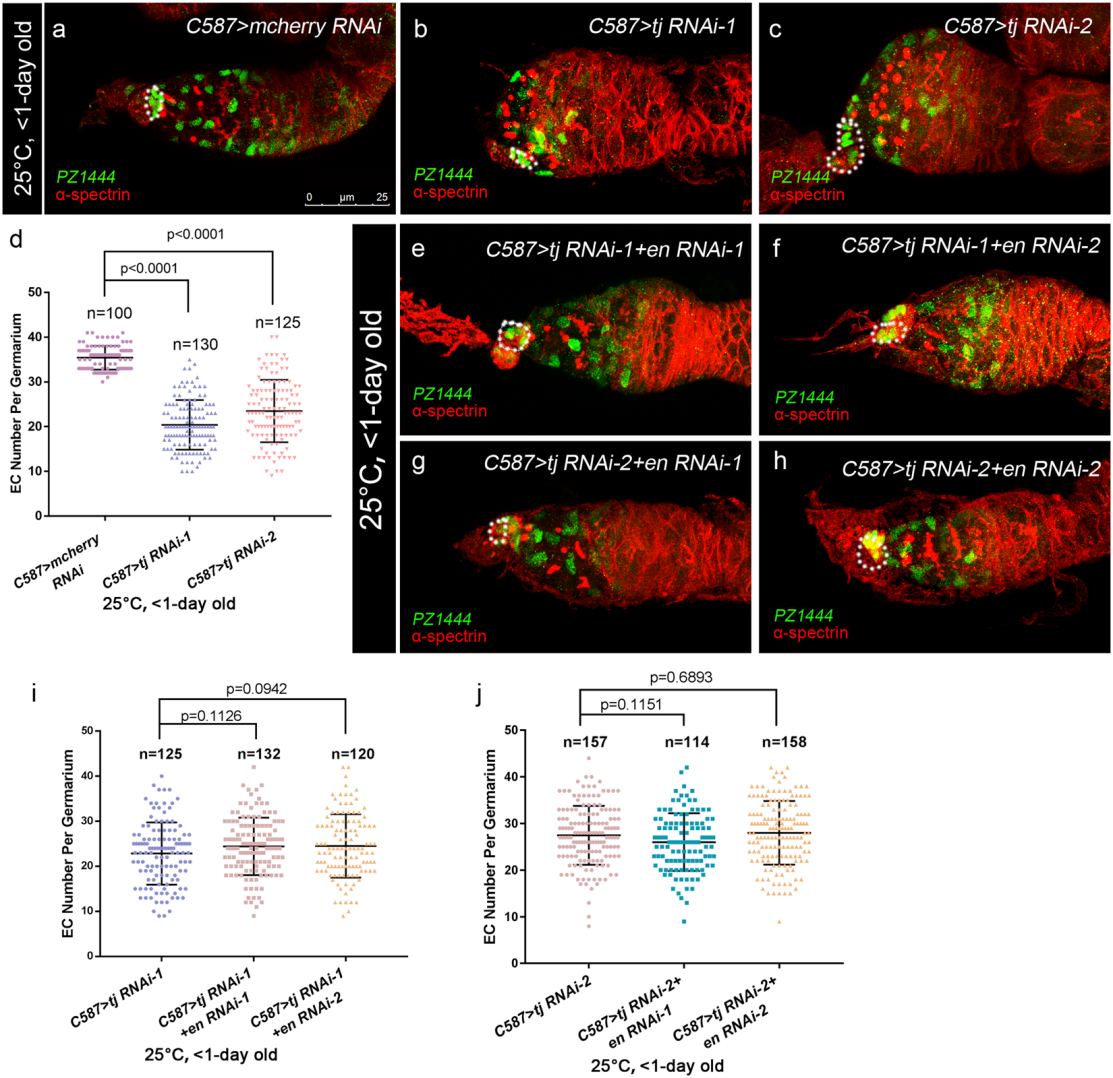
Tj regulates EC number independent of En. **(a–c,e–h)** Germaria from newly born (<1-day old) females are stained for α-spectrin (red), to mark spectrosomes and fusomes. The *PZ1444*/+ is introduced to indicate ECs and CpC (marked by β-gal, green). White broken ovals indicate CpC clusters. The control group **(a)** exhibits a normal EC number. The *C587* > *tj RNAi-1* **(b)** or *C587* > *tj RNAi-2* **(c)** groups exhibit a reduction of EC number. **(d)** Graph shows the ECs number in germaria of indicated genotypes. Error bars are shown as Means ± S.D. of each genotype. The *C587* > *tj RNAi-1* + *en RNAi-1* **(e)** and *C587* > *tj RNAi-1* + *en RNAi-2* **(f)** groups show a similar EC number to *C587* > *tj RNAi-1* **(b)**. The *C587* > *tj RNAi-2* + *en RNAi-1* **(g)** *and C587* > *tj RNAi-2* + *en RNAi-2* **(h)** groups show a similar EC number to *C587* > *tj RNAi-2* **(c)**. **(i,j)** Graphs show the ECs number in germaria of indicated genotypes. Error bars are shown as Means ± S.D. of each genotype. Scale bar is showed in panel (a). Anterior is always to the left.

Previous studies have also shown that UGC accumulation caused by extrinsic EC defects is always coupled with severe loss of ECs^10,24–26,28,46^, indicating that a certain number of EC is required for non-cell autonomous control of germline differentiation. To determine whether Tj controls EC number, the enhancer trap line *PZ1444*^52,53^, which labels both CpCs and ECs, was used to directly quantify the EC number in germaria. The crosses were set up at 25 °C and newly eclosed females (<1-day old) were dissected for detection. In germaria of control group (Fig. 9a), *PZ1444* is expressed higher in CpCs than in ECs. ECs also differ from CpCs by location and the nuclear morphology. In contrast, *C587*-mediated *tj* KD led to a significant reduction of β-gal positive ECs (Fig. 9b–d). Furthermore, the *lacZ* expression was dampened in ECs upon *tj*-depletion. Pupal-specific *tj* KD with *C587*^*ts*^ induced consistent phenotype (Fig. S8j). Surprisingly, no significant reduction in EC number was detected in the adult-specific regimens for *C587*^*ts*^-mediated *tj* KD (Fig. S8k). Together with observations on the small EC clone size for *tj* mutant (Figs 1g,h and S8g–i), these pieces of evidence lead to a conclusion that Tj is required in pre-adult stage for the establishment of a proper EC number, which may contribute to proper GSC progeny differentiation.

To test whether Tj regulates EC cell number through En, *en* RNAi and *tj* RNAi were coexpressed using *C587-GAL4* at 25 °C. No rescue of EC number was observed following co-expression of *en* RNAi in newly eclosed (<1-day old) females, suggesting that En is dispensable for *tj*-mediated regulation of EC number (Fig. 9e–j).

## Discussion

Results from earlier studies^33,37^ and ours have shown that Tj absence as induced by a strong loss-of-function allele has deleterious effects on ovariole formation and maintenance. Here we generate a hypomorphic condition of Tj knockdown using *C587-GAL4*-mediated *tj RNAi*, and show that Tj is required in pre-adult ECs for CB differentiation control. In our hands, the GSC progeny differentiation process was not disrupted upon *hh*-mediated *tj* KD, though it could be due to an incomplete Tj depletion in CpCs. However, our molecular and genetic studies reveal that *tj* KD drives UGC formation, possibly through an En-dependent mechanism of repressing *dpp* transcription outside the GSC niche. Tj also intrinsically maintains EC protrusions and EC number in pre-adult stage independent of En. Therefore, we propose that Tj functions to safeguard against the acquisition of GSC niche cell characteristics by immature ECs and promote pre-adult EC to adopt the identities of differentiation niche cells.

Strikingly, UGCs forming upon *tj* depletion express *bamP-GFP*, which is similar to some previous studies^10,12,54^. Furthermore, *bamP-GFP*-positive UGCs are considered as CB tumors, and in a recent study they are believed to have been already escaped niche signal^15^. Remarkably, our data support the idea that the accumulated CB-like cell is probably resulting from an ectopic BMP activity sustained at a certain level outside the GSC niche, as evident by the supernumerary pMad-negative *dad-lacZ-*positive UGCs in *C587-*mediated *tj RNAi* germaria. Meanwhile, the elevated *dpp* transcription in *tj*-depleted ECs was also confirmed with two distinct enhancer traps, *dpp2.0-lacZ* and *P4-lacZ*. As a consequence of ectopic BMP activity, the amount of Bam protein failed to reach the critical level for 2-cell cyst formation. Taken together with the observation that *hs-bam* rescued *tj* differentiation phenotype, we suggest a direct link between CB-like cell accumulation and disreugulated BMP activity.

What motivates the ectopic BMP activity outside the GSC niche? Several correlative studies suggest complex mechanisms, in which a population of ECs is capable of expressing Dpp as well as other niche cell markers under certain conditions^20,21,25,26,28^. Together, they reveal that tightly regulated *dpp* transcription is required to maintain homeostasis of adult ovaries. Our study highlights the role of Tj required for modulating *dpp* transcription activity partly through repressing En expression during ovarian development. This suggests that, in wild-type developing ovaries, the enhancer element dpp2.0 is maintained in an inactive state in most of the ECs by Tj-mediated repression of En, an activator of the enhancer element^29^. How disrupted Tj expression leads to upregulation of En remains an open question. Meanwhile, how the enhancer element P4 participate in the Tj-involved regulation of *dpp* transcription remains to be uncovered. Thus, further studies investigating the regulatory network of *dpp* transcription would be of great interest.

Our observation that *tj*-depleted ECs exhibit a dual identity, simultaneously harboring features of CpCs and ECs, particularly recalls the previous observation that Tj functions in CpCs for TF fate repression^37^. Thus, it is reasonable that different expression levels of Tj^37^ can be instructive for the cellular differentiation status of TF, CpCs and ECs. Meanwhile, these observations also explain why the immunostaining of En in *C587* > *tj RNAi* CpCs or *tj*^*PL3*^ clonal CpCs (Fig. S5j–j”’) appeared to be visibly unchanged. (It is still possible that the upregulated En expression has been below our detection threshold.) Due to adoption of TFC fate, *tj*-depleted CpCs exhibit comparable En staining to TFCs, in which En staining is normally indistinguishable from CpCs. And here, we consider Tj as a factor that regulates the balance between stem cell and differentiation niches so as to coordinate the behavior or GSC and GSC progeny during development.

On the other hand, slightly extra UGCs without inappropriate En expression were uncovered by MARCM analysis, probably owing to the small clone size of *tj* mutant. Accordingly, we argue that Tj helps to establish a certain number of ECs. Consistently, *PZ1444* reporter analysis with pupal-specific *tj* KD gave the similar conclusion. Meanwhile, we found altered clonal EC morphology in the MARCM analysis (Fig. S8g–i, white arrows). Notably, RNAi based pupal-specific depletion of Tj caused a severe loss of EC protrusions. These results are consistent with the previous study that the presence of wild-type ECs is sufficient to partially rescue the phenotype induced by *woc* or *stat* mutant ECs^15^. The tight association between germline and ECs is indispensable for proper germline differentiation^10,23–25,46^. Tj has been reported to participate in the interaction between PGCs and ICs^33,37^. Here we further identify that Tj is critical for protrusions of ECs in a cell-autonomous manner. As EC morphology was not regained when germline was forced to differentiate in *tj* KD germaria. Together with the observation that *tj* phenotype of EC number and cellular protrusion were not rescued upon *tj en* double knock down, our data strongly suggest that Tj controls germline differentiation via multiple types of mechanism, including the ones dependent and independent of En.

Besides, Tj appears to function primarily during the developmental stage rather than adulthood. As the En-dependent and independent roles of Tj in CB differentiation control are mainly played during the pupal stage. In this context, a better understanding of hormones, developmental cues and chromatin accessibility is essential for understanding the normal roles of Tj in development.

## Methods

### Fly strains and genetics

All *Drosophila* strains were maintained at 25 °C on standard cornmeal media supplied with live yeast unless otherwise stated. The following fly stocks were used in this study: *y, w* (BDSC 6598), *tj*^*Δ1*^ (null allele^55^, gift from Dorothea Godt), *tj*^*PL3*^ (null allele^33^, BDSC 4987), *y, w, hs-Flp, UAS-mCD8-GFP; tubP-GAL80, FRT40A; tubP-GAL4* (BDSC 42725), *FRT40A, Ubi-GFP* (BDSC 5189), *FRT40A* (BDSC 8212), *C587-GAL4* (gift from Yu Cai), *hh-GAL4* (gift from Xinhua Lin), *tj-GAL4* (DGRC 104034), *tub-GAL80*^*ts*^ (BDSC 7017, 7019), *C587-GAL4, UAS-Flp* (gift from Cai Yu), *tj* RNAi-1(BDSC 25987), *tj* RNAi-2(BDSC 34595), other *tj* RNAi lines in Supplymentary(VDRC 30525, 30526, 108255), *dpp* RNAi-1 (BDSC 25782), *dpp* RNAi-2 (BDSC 31530), *en* RNAi-1 (VDRC 105678), *en* RNAi-2 (BDSC 33715), *mcherry* RNAi (BDSC 35785), *UAS-dpp* (BDSC 1486), *dad-lacZ* (gift from Yu Cai), *bamP-GFP* (gift from Dennis M. McKearin), *dpp*^*hr4*^ (gift from Ting Xie), *dpp*^*e90*^ (gift from Ting Xie), *dpp2.0-lacZ* (gift from Yu Cai), *P4-lacZ* (gift from Rongwen Xi), *en*^4^ (BDSC 1817), *en*^7^ (BDSC 1820), *hh-lacZ* (BDSC 5330), *Dl-lacZ* (*Dl*^05151^, BDSC 11651).

### Larval and pupal staging

Staging of larvae and pupae was performed as reported^40,41,56^. In short, synchronized eggs were collected in a fresh vial within 2 hours. After the parents were removed, the offspring were cultured with optimal nutrition and uncrowded conditions. For flies without *tub-Gal80*^*ts*^, the vials were kept at 25 °C all the time. Under these conditions, the middle of larval third instar (ML3) is at 96 h after egg laying (AEL), the late of larval third instar (LL3) is between 114–116 h AEL, and larval-to-pupal transition (LL3 to white pre-pupa) is at 120 h AEL. The early pupal (EP) is at 144 h AEL. For flies that carry *tub-Gal80*^*ts*^, vials were kept at 18 °C until temperature shift. Under these conditions, the LL3 is 9 days AEL, and the EP is 12 days AEL.

### Heat shock and clone generation

MARCM clone of *tj* mutant and control were generated by crossing *FRT40A, tj*^*PL3*^ or *FRT 40 A* with *y, w, hs-Flp, UAS-mCD8-GFP; tubP-GAL80, FRT 40A; tubP-GAL4*. The resulting larvae were heat shocked at 37 °C with three 60 min pulses at 5 h intervals each day since ML3 to EP. After heat shock, the flies were kept at 29 °C until dissection. Newly born (<1-day old) females were dissected and stained with the appropriate antibodies.

Cap cell clones were generated by crossing *FRT40A, tj*^*PL3*^ or *FRT 40A* with *C587-GAL4, UAS-Flp; FRT40A GFP* at 25 °C. The resulting larvae were kept at 29 °C until eclosion. Newly born (<1-day old) females were dissected and stained with the appropriate antibodies.

Flies carrying *hs-bam* were heated shocked at 37 °C for 60 min at the 8^th^ day AEL. Newly born (<1-day old) females were dissected and stained with the appropriate antibodies.

### Antibodies and immunofluorescence

The staining of ovaries or larvae gonads or pupae gonads was carried out as described previously^57,58^. In brief, females were dissected in PBS and fixed in 4% paraformaldehyde (Sigma) in PBS for 30 min, rinsed with 0.3% PBST [PBS containing 0.3% Triton X-100 (Bio-Rad)] three times, permeated with 1.0% PBST for 1 h, blocked with 10% goat serum (Life Technology) in 0.3% PBST for 2 h, and stained overnight at 4 °C with primary antibodies. Ovaries or gonads were then washed three times in 0.3% PBST and incubated with secondary antibodies for 2 h at room temperature and stained with DAPI for 10 minutes. Finally, ovaries were rinsed four times with 0.3% PBST and mounted.

The following primary antibodies were used in this work: Mouse anti-α-spectrin (1:20, DSHB 3A9), rabbit anti-Vasa (1:200 Santa Cruz), rat anti-Vasa (1:10 DSHB), mouse anti-β-gal (1:500, Promega), rabbit anti-β-gal (1:1000, Cappel), guinea pig anti-Tj (1:1000, gift from D. Godt), rabbit anti-Zfh1 (1:1000, gift from R. Lehmann), rabbit anti-pMad (1:1000, gift from E. Laufer), rabbit anti-GFP (1:1000, Life Technology), mouse anti-Engrailed (1:10, 4D9 DSHB), mouse anti-Lamin C (1:10, LC28.26 DSHB), mouse anti-Coracle (1:10, C615.16 DSHB). Secondary antibodies conjugated with Alexa Fluor 488, 546 or 647 (Life Technology) were used at 1:1000 dilutions. DAPI (Life Technology) was used to visualize the nuclei. Confocal images were captured on Leica TCS SP8 laser confocal microscope and obtained using the Leica AF Lite system. Images were processed in Adobe Photoshop CS6.

### RNA extraction and qRT-PCR

RNA was isolated from newly eclosed female ovaries of *tj-GAL4* > *mcherry RNAi*, *tj- GAL4* > *tj RNAi-1* and *tj- GAL4* > *tj RNAi-2*. And *rp49* was served as normalization control. To exclude the interference of the vitellarium region of ovariole, we chose the female eclosed within 2 h for dissection. RNA was isolated using TRIzol (Invitrogen) and then subjected to reverse transcription. ReverTra Ace® qPCR RT Master Mix with gDNA Remover (TOYOBO) was used for cDNA synthesis according to the manufactures’ instructions. Quantitative real-time PCR was performed on Bio-Rad CFX96 PCR system by using SYBR green qPCR Master Mix (TOYOBO).

The primers used for amplifying *dpp* and *rp49* mRNA are as follows:

*dpp* forward: AGCCGATGAAGAAGCTCTACG

*dpp* reverse: ATGTCGTAGACAAGCACCTGGTA

*rp49* forward: TCCTACCAGCTTCAAGATGAC

*rp49* reverse: CACGTTGTGCACCAGGAACT

Relative concentration was determined using the 2^−ΔΔCt^ method in Bio-Rad CFX Manager Software Ver 3.0.

### Statistical analysis

All statistical data were recorded in Excel (Microsoft) and graphed in Prism 7.0 (GraphPad Software). The Chi-square (and Fisher’s exact) test and the two-tailed Student’s *t*-test were used to calculate the p-values.

### Data Availability

All data generated or analyzed during this study are included in this published article (and its Supplementary Information files).

## Supplementary information

supplementary materials
