# Supplementary material for: Traffic jam regulates the function of the ovarian germline stem cell progeny differentiation niche during pre-adult stage in Drosophila
Source: Sci Rep. 2019 Jul 12;9:10124. doi: 10.1038/s41598-019-45317-6 (PMC6626045; doi:10.1038/s41598-019-45317-6)
Supplement: Supplementary file 1 — supplementary materials [file 41598_2019_45317_MOESM1_ESM.pdf]

Supplementary Information

Traffic jam regulates the function of the ovarian germline stem cell progeny differentiation niche during pre-adult stage in *Drosophila*

Mengjie Li[1], Xiaolong Hu[1], Shu Zhang[2], Margaret S. Ho[4], Geng Wu[1*] and Lei Zhang[3, 4 *]

1 State Key Laboratory of Microbial Metabolism, School of Life Sciences & Biotechnology, The Joint International Research Laboratory of Metabolic & Developmental Sciences, Shanghai Jiao Tong University, Shanghai, China, 200240, China

2 School of Life Sciences and Biotechnology, Shanghai Jiao Tong University, Shanghai, 200240, China

3 State Key Laboratory of Cell Biology, CAS Center for Excellence in Molecular Cell Science, Innovation Center for Cell Signaling Network, Shanghai Institute of Biochemistry and Cell Biology, Chinese Academy of Sciences, University of Chinese Academy of Sciences, Shanghai, 200031, China.

4 School of Life Science and Technology, Shanghai Tech University, Shanghai, 201210, China

* corresponding. rayzhang@sibcb.ac.cn
geng.wu@sjtu.edu.cn

Figure S1

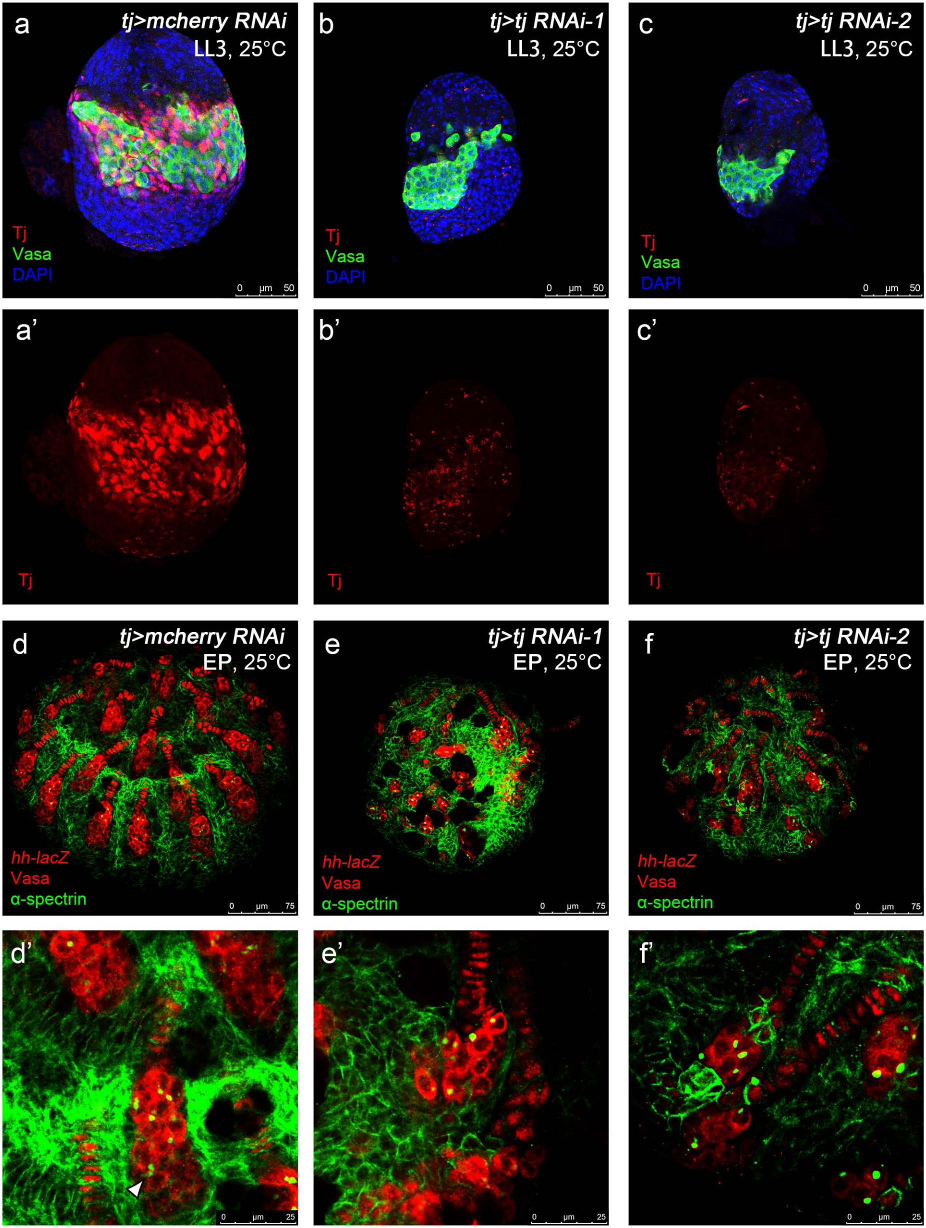
a
tj>mcherry RNAi
LL3, 25°C
Tj
Vasa
DAPI
0 µm 50
b
tj>tj RNAi-1
LL3, 25°C
Tj
Vasa
DAPI
0 µm 50
c
tj>tj RNAi-2
LL3, 25°C
Tj
Vasa
DAPI
0 µm 50
a'
Tj
b'
Tj
c'
Tj
d
tj>mcherry RNAi
EP, 25°C
hh-lacZ
Vasa
α-spectrin
0 µm 75
e
tj>tj RNAi-1
EP, 25°C
hh-lacZ
Vasa
α-spectrin
0 µm 75
f
tj>tj RNAi-2
EP, 25°C
hh-lacZ
Vasa
α-spectrin
0 µm 75
d'
0 µm 25
e'
0 µm 25
f'
0 µm 25

Figure S1. The transgenic RNAi lines against *tj* work efficiently.

(a-c') LL3 female gonads are stained for Tj (red), to mark Tj expression, Vasa (green), to mark germline. DAPI is blue. In control group **(a, a')**, Tj protein is concentrated in ICs, which mix uniformly with PGCs. In *tj>tj RNAi-1* **(b, b')** and *tj >tj RNAi-2* **(c, c')** groups, Tj protein is eliminated efficiently. **(d-f')** EP female gonads are stained for α-spectrin (green), to mark spectrosomes and fusomes, Vasa (red), to mark germline. The *hh-lacZ/+* is introduced to label the TF and CpCs (marked by β-gal, red). The control gonad **(d, d')** is well-organized in primordial ovarioles. And each ovariole exhibits differentiating germline cysts with branched fusomes, as indicated with white arrowhead. The *tj>tj RNAi-1* **(e, e')** and *tj>tj RNAi-2* **(f, f')** groups exhibit disorganized primordial ovarioles and lack branched fusome-containing cysts, which phenocopy *tj* mutant gonads. Scale bar is shown in each panel. Anterior is up.

Figure S2

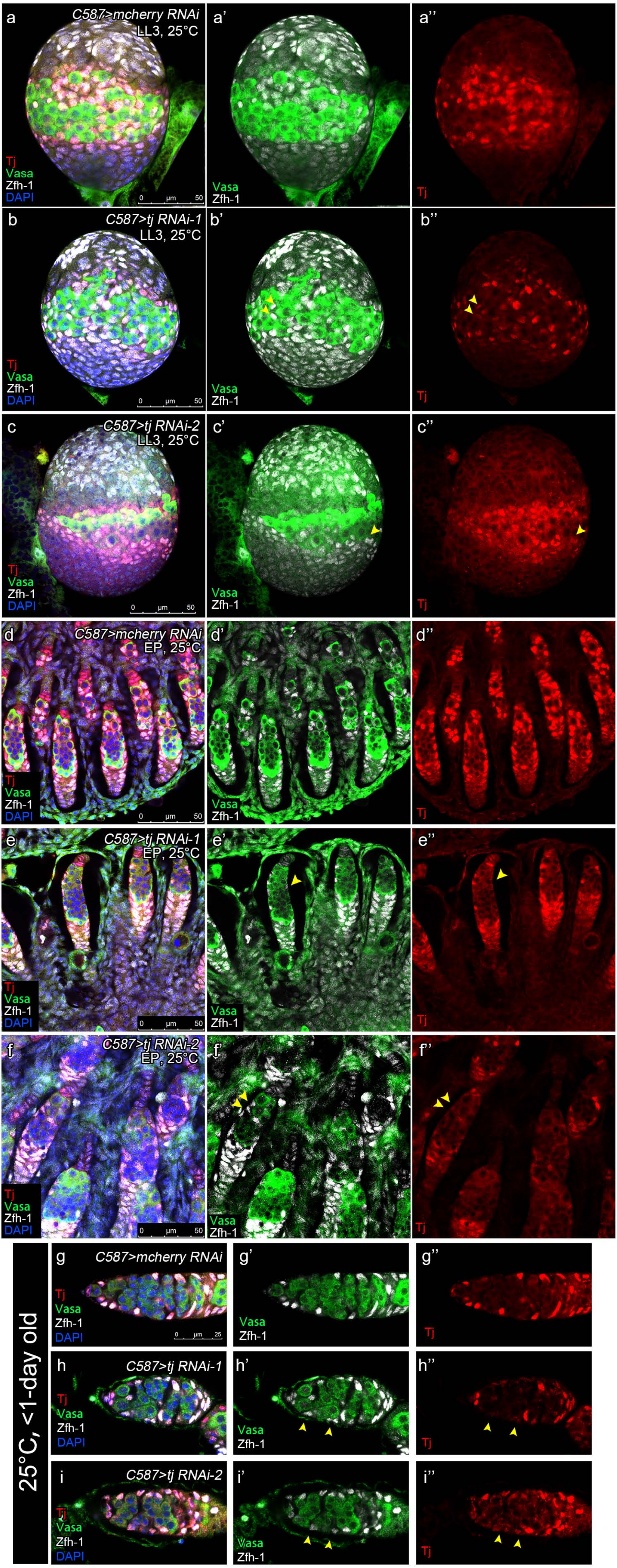
a
C587>mcherry RNAi
LL3, 25°C
a'
a''
Tj
Vasa
Zfh-1
DAPI
0 µm 50
b
C587>tj RNAi-1
LL3, 25°C
b'
b''
c
C587>tj RNAi-2
LL3, 25°C
c'
c''
d
C587>mcherry RNAi
EP, 25°C
d'
d''
e
C587>tj RNAi-1
EP, 25°C
e'
e''
f
C587>tj RNAi-2
EP, 25°C
f'
f''
25°C, <1-day old
g
C587>mcherry RNAi
g'
g''
0 µm 25
h
C587>tj RNAi-1
h'
h''
i
C587>tj RNAi-2
i'
i''

Figure S2. *C587*-driven *tj* RNAi lines reduce Tj expression with low efficiency in IC/ECs.

(a-i'') Female gonads or ovaries are stained for Tj (red), to mark Tj expression level, Zfh-1 (white), to mark somatic cells, Vasa (green), to mark germline. DAPI is blue. **(a-c'')** LL3 female gonads exhibit a uniform mixture of ICs and PGCs. In control group **(a-a'')**, Tj protein is concentrated in ICs, which are also labeled with Zfh-1. The *C587>tj RNAi-1* **(b-b'')** and *C587>tj RNAi-2* **(c-c'')** groups display faint staining of Tj in some of the ICs, which show strong Zfh-1 staining, as indicated by yellow arrowheads. **(d-f'')** EP female gonads exhibit well-organized primordial ovarioles. In control group **(d-d'')**, Tj protein is concentrated in CpCs and pre-adult ECs, which are also labeled with Zfh-1. In *C587>tj RNAi-1* **(e-e'')** and *C587>tj RNAi-2* **(f-f'')** groups, Tj protein is eliminated in some of the pre-adult ECs, which exhibit strong Zfh-1 staining, as indicated by yellow arrowheads. **(g-i'')** Germaria from newly eclosed (<1-day old) females are examined. Zfh-1 is highly expressed in somatic cells. Control group **(g-g'')** presents intensive staining of Tj in somatic cells, excluding TF. *C587>tj RNAi-1* **(h-h'')** and *C587>tj RNAi-2* **(i-i'')** groups exhibit elimination of Tj in some of the ECs, as indicated by yellow arrowheads. Scale bar for (a-f'') is shown in each panel. Scale bar for (g-i'') is shown in (g). Anterior is up in (a-f'') and to the left in (g-i'').

Figure S3

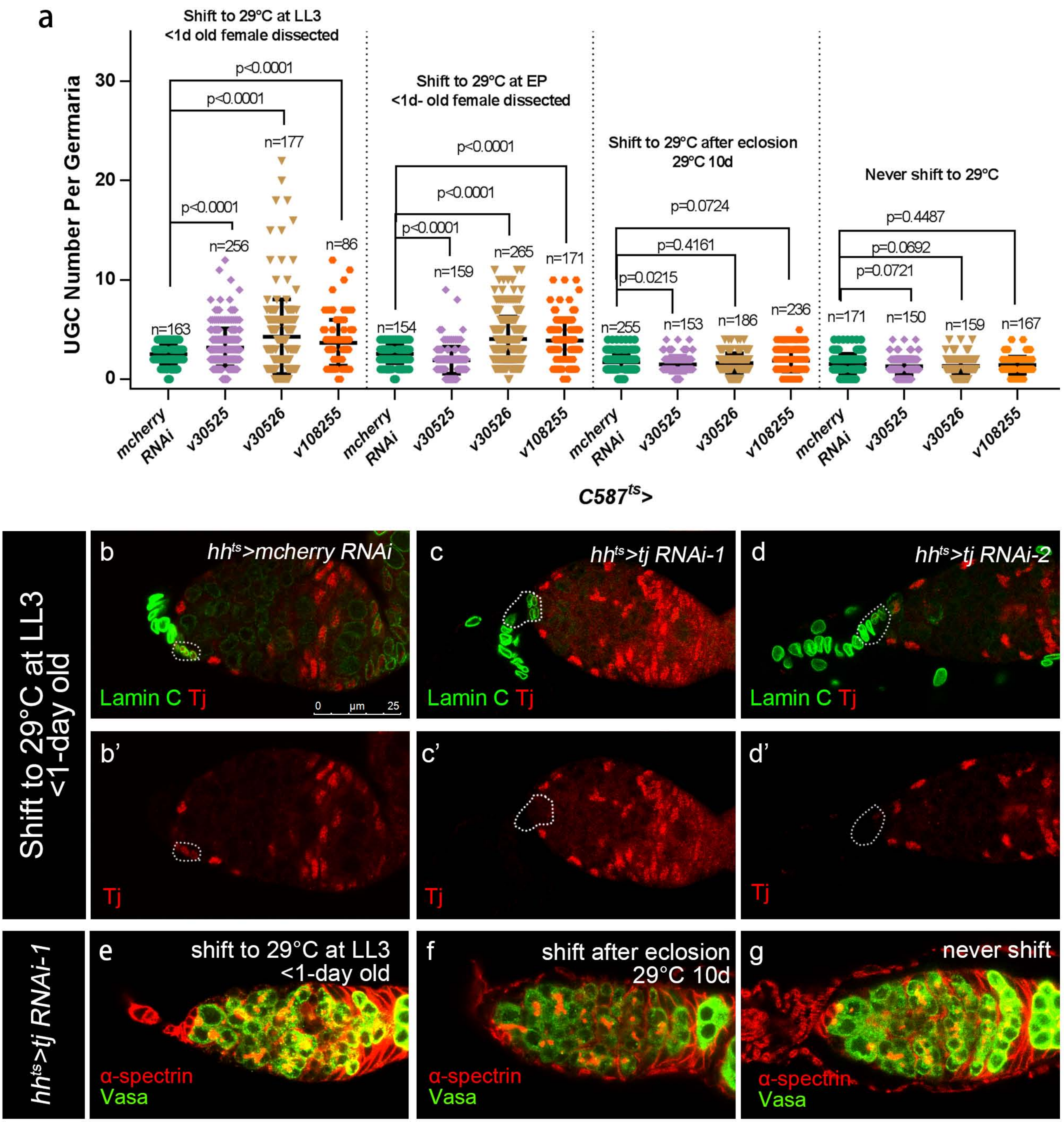

a
Shift to 29°C at LL3
<1d old female dissected
Shift to 29°C at EP
<1d- old female dissected
Shift to 29°C after eclosion
29°C 10d
Never shift to 29°C
UGC Number Per Germaria
p<0.0001
p<0.0001
p<0.0001
p=0.0724
p=0.4161
p=0.0215
p=0.4487
p=0.0692
p=0.0721
n=163
n=256
n=177
n=86
n=154
n=159
n=265
n=171
n=255
n=153
n=186
n=236
n=171
n=150
n=159
n=167
mcherry RNAi
v30525
v30526
v108255
C587ts>
b
hhts>mcherry RNAi
c
hhts>tj RNAi-1
d
hhts>tj RNAi-2
Lamin C Tj
Tj
b'
c'
d'
Shift to 29°C at LL3
<1-day old
hhts>tj RNAi-1
e
shift to 29°C at LL3
<1-day old
f
shift after eclosion
29°C 10d
g
never shift
α-spectrin
Vasa

Figure S3. *C587*-mediated *tj* RNAi induces excess UGC accumulation in pre-adult stage and *hh*-mediated *tj* RNAi reduce Tj expression in CpCs but does not induce excess UGCs.

(a) Graph shows the UGCs number for each genotype as indicated. Error bars are shown as Means ± S.D. of each genotype. $C587^{ts}$-driven other *tj* RNAi lines induce a similar phenotype to *tj RNAi-1* and *tj RNAi-2*. **(b-d')** Females from hh^{ts}-driven *tj* RNAi are raised at 18°C up to LL3 and then transferred to 29°C. Germaria from newly eclosed (<1-day old) females are stained for Tj (red), to mark Tj expression, Lamin C (green), to mark TF and CpCs. White open ovals indicate CpCs. In control group **(b, b')**, Tj is expressed in CpCs as well as in ECs. In hh^{ts}>*tj RNAi-1* **(c, c')** and hh^{ts}>*tj RNAi-2* **(d, d')** groups, Tj protein is almost diminished in cap cells. **(e-g)** Germaria from hh^{ts}>*tj RNAi-1* females are stained for α-spectrin (red), to mark spectrosomes and fusomes, Vasa (green), to mark germline. hh^{ts}>*tj RNAi-2* presents similar phenotype and the data is not shown. Germaria from newly eclosed flies (<1-day old), which are raised at 18°C up to LL3 and then maintained at 29°C before dissection **(e)**, and germaria from 10-day-old females, which are raised at 18°C up to adulthood and then maintained at 29°C **(f)**, present normal CBs number, which are similar to the flies never shifted to 29°C **(g)**. Scale bar is shown in (b). Anterior is always to the left.

Figure S4

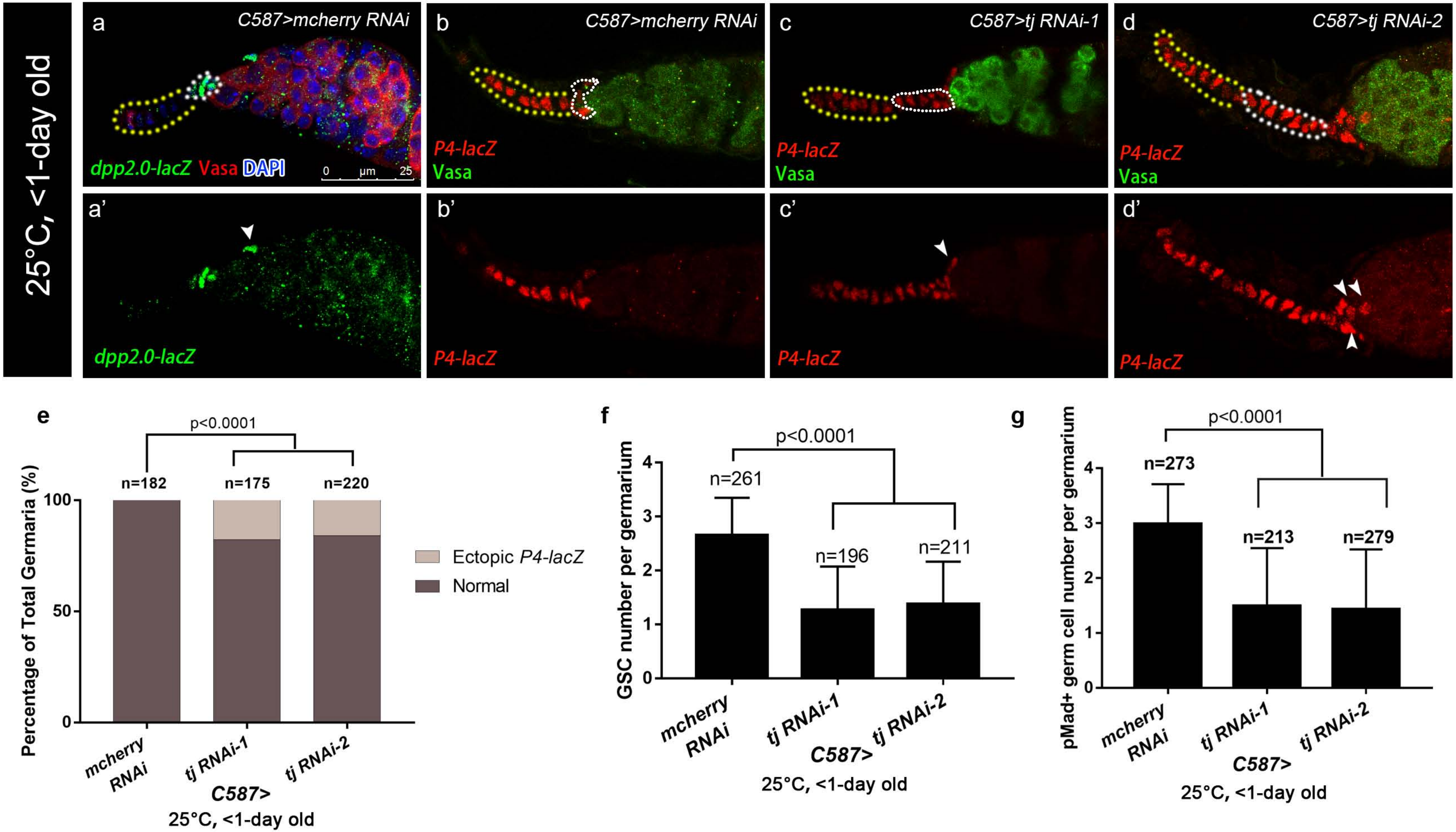
25°C, <1-day old
a
C587>mcherry RNAi
dpp2.0-lacZ Vasa DAPI
0 μm 25
b
C587>mcherry RNAi
P4-lacZ
Vasa
c
C587>tj RNAi-1
P4-lacZ
Vasa
d
C587>tj RNAi-2
P4-lacZ
Vasa
a'
dpp2.0-lacZ
b'
P4-lacZ
c'
P4-lacZ
d'
P4-lacZ
e
p<0.0001
n=182
n=175
n=220
Percentage of Total Germaria (%)
100
50
0
Ectopic P4-lacZ
Normal
mcherry RNAi
tj RNAi-1
tj RNAi-2
C587>
25°C, <1-day old
f
p<0.0001
n=261
n=196
n=211
GSC number per germarium
4
3
2
1
0
mcherry RNAi
tj RNAi-1
tj RNAi-2
C587>
25°C, <1-day old
g
p<0.0001
n=273
n=213
n=279
pMad+ germ cell number per germarium
4
3
2
1
0
mcherry RNAi
tj RNAi-1
tj RNAi-2
C587>
25°C, <1-day old

Figure S4. *C587*-mediated *tj* RNAi induces ectopic *dpp* in ECs and decreased GSC number.

(a, a') Germaria from newly eclosed flies (<1-day old) are stained for Vasa (red) to mark germline. DAPI is blue. The *dpp2.0-lacZ/+* is introduced to visualize the *dpp* transcription (marked by β-gal, green). In control group, about 12% of the total germaria show *dpp2.0-lacZ* expression in some ECs, as indicated by white arrowhead. **(b-d')** Germaria from newly eclosed flies (<1-day old) are stained for Vasa (green), to mark germline. The *P4-lacZ/+* is introduced to visualize the *dpp* transcription (marked by β-gal, red). Yellow dashed lines indicate TFs and white open ovals indicate CpC clusters. The control group **(b)** displays an exclusive expression of *P4-lacZ* in TF and CpCs. The *C587>tj RNAi-1* **(c)** and *C587>tj RNAi-2* **(d)** groups display ectopic *P4-lacZ* expression in ECs, as indicated by white arrowheads. **(e)** Graph shows the percentage of germaria that contain ectopic *P4-lacZ* expressions in ECs for each genotype. **(f)** Graph shows the GSC number for each genotype shown in Figure 3(a-c). Data are shown as Means ± S.D. of each genotype. Knocking down of *tj* with *C587-GAL4* causes reduced GSC number in comparison to the control germaria. **(g)** Graph shows the number of pMad-positive germ cells for each genotype shown in Figure 3(a-c). Data are shown as Means ± S.D. of each genotype. *C587*-mediated *tj* RNAi causes pMad-negative UGCs. Indeed, the pMad-positive germ cell number is reduced in *tj* KD germaria compared with that in control. Scale bar is shown in (a). Anterior is always to the left.

Figure S5

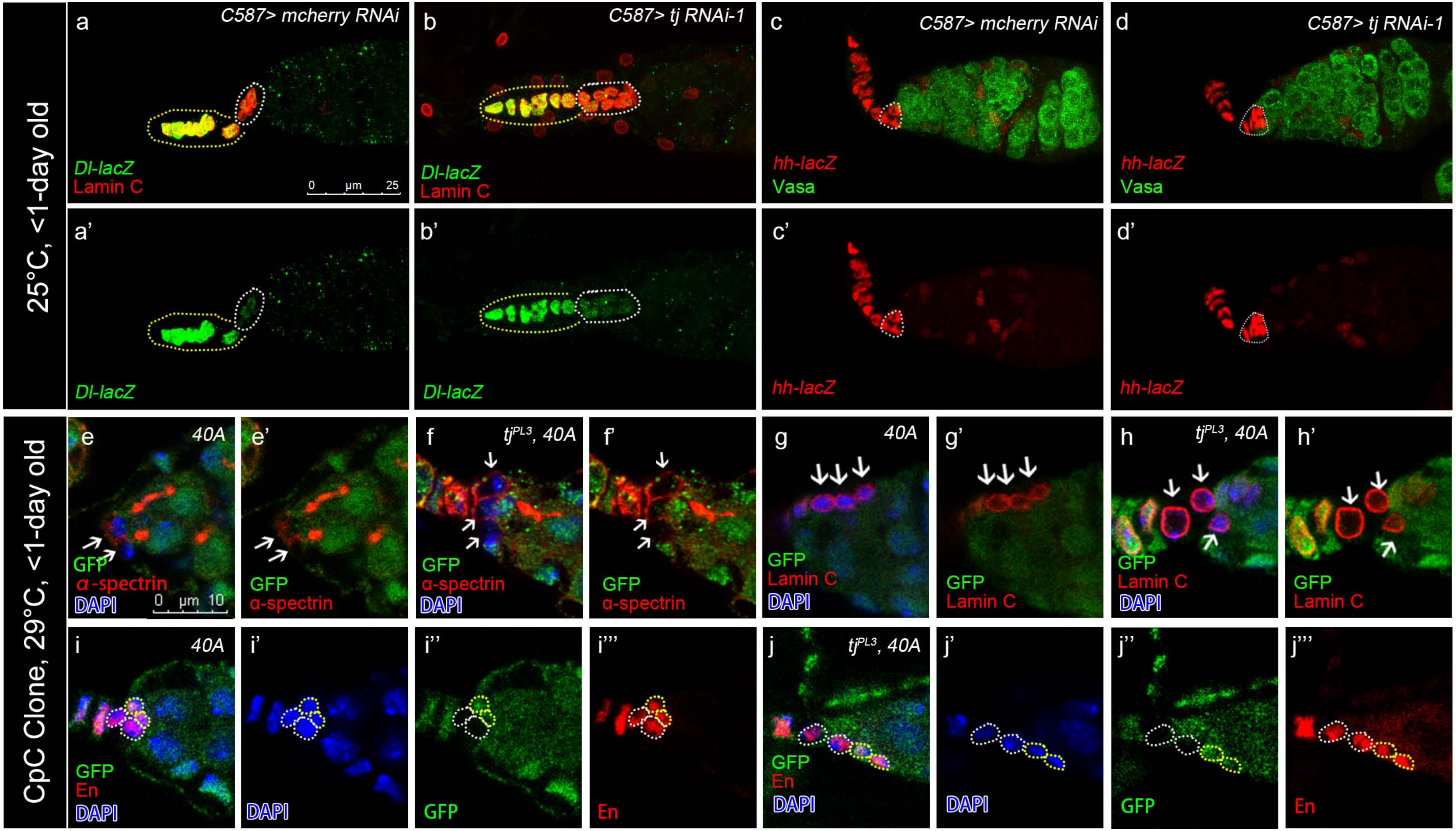

25°C, <1-day old
a
C587> mcherry RNAi
Dl-lacZ
Lamin C
0 μm 25
b
C587> tj RNAi-1
Dl-lacZ
Lamin C
c
C587> mcherry RNAi
hh-lacZ
Vasa
d
C587> tj RNAi-1
hh-lacZ
Vasa
a'
Dl-lacZ
b'
Dl-lacZ
c'
hh-lacZ
d'
hh-lacZ
CpC Clone, 29°C, <1-day old
e
40A
GFP
α-spectrin
DAPI
0 μm 10
e'
GFP
α-spectrin
f
tj^PL3, 40A
GFP
α-spectrin
DAPI
f'
GFP
α-spectrin
g
40A
GFP
Lamin C
DAPI
g'
GFP
Lamin C
h
tj^PL3, 40A
GFP
Lamin C
DAPI
h'
GFP
Lamin C
i
40A
GFP
En
DAPI
i'
DAPI
i''
GFP
i'''
En
j
tj^PL3, 40A
GFP
En
DAPI
j'
DAPI
j''
GFP
j'''
En

Figure S5. *C587*-mediated *tj* knockdown does not induce ectopic TFCs and CpCs.

(a-b') Germaria from newly eclosed flies (<1-day old) are stained for Lamin C (red) to mark TF and CpCs. The enhancer trap line *Dl-lacZ/+* is introduced to indicate TFs (marked by β-gal, green). Yellow dashed lines indicate TFs. White broken ovals indicate CpCs. In the control germaria **(a, a')**, *Dl-lacZ* is high in TF, lower in some CpCs and undetectable in the other CpCs. There in an average of 7-8 TFCs in a germarium. And the *C587>tj RNAi-1* group **(b, b')** contains a similar number of TFCs as control group. **(c-d')** Germaria from newly eclosed flies (<1-day old) are stained for Vasa (green) to mark germline. The enhancer trap line *hh-lacZ/+* is introduced to indicate TF and CpCs (marked by β-gal, red). White broken ovals indicate CpCs. The control group **(c, c')** shows high expression of *hh-lacZ* in both TFs and cap cells and low expression in ECs. The *C587>tj RNAi-1* **(d, d')** group shows no alteration of *hh-lacZ* expression in ECs. *C587>tj RNAi-2* group exhibits a similar pattern to *C587>tj RNAi-1* and the data is not shown. **(e-j''')** Germaria from newly born (<1-day old) mosaic females are stained for GFP (green), to mark non-recombined cells (wild-type cells). The control and *tj* mutant CpC clones are recognized by the absence of GFP. (**e-f'**) α-spectrin (red) marks spectrosomes, fusomes and somatic cell cortical cytoskeleton. White arrows indicate clonal cells. **(g-h')** Lamin C (red) marks TF and CpCs. White arrows indicate clonal cells. **(i-j''')** En (red) marks TF and CpCs. White open ovals indicate clonal CpCs and yellow open ovals indicate non-clonal CpCs. Mosaic germaria of control **(e, e', g, g', i-**

i''') and *tj* mutant **(f, f', h, h', j-j''')** exhibit a similar distribution of number of -GFP-negative cap cells. Scale bar for (a-d') is shown in (a). Scale bar for (e-j''') is shown in (e). Anterior is always to the left.

Figure S6

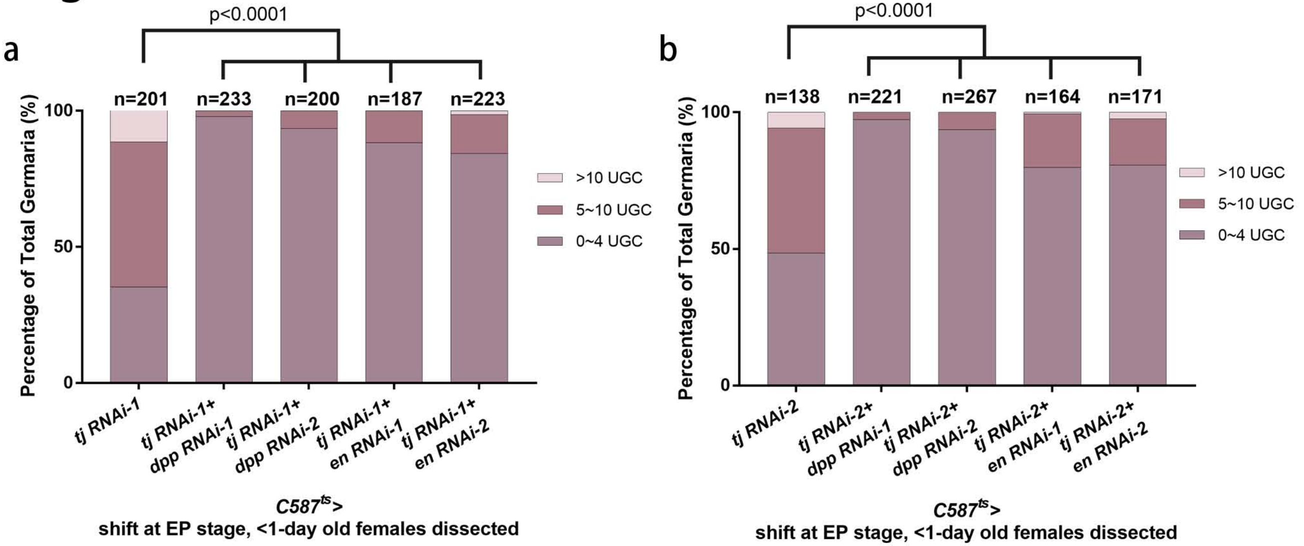
a
p<0.0001
n=201
n=233
n=200
n=187
n=223
Percentage of Total Germaria (%)
100
50
0
>10 UGC
5~10 UGC
0~4 UGC
tj RNAi-1
tj RNAi-1+ dpp RNAi-1
tj RNAi-1+ dpp RNAi-2
tj RNAi-1+ en RNAi-1
tj RNAi-1+ en RNAi-2
C587ts>
shift at EP stage, <1-day old females dissected
b
p<0.0001
n=138
n=221
n=267
n=164
n=171
Percentage of Total Germaria (%)
100
50
0
>10 UGC
5~10 UGC
0~4 UGC
tj RNAi-2
tj RNAi-2+ dpp RNAi-1
tj RNAi-2+ dpp RNAi-2
tj RNAi-2+ en RNAi-1
tj RNAi-2+ en RNAi-2
C587ts>
shift at EP stage, <1-day old females dissected

Figure S6. *C587^{ts}*-mediated pupal-specific *tj dpp* or *tj en* double knockdown significantly repress the *tj* KD UGC phenotype.

(a, b) Graphs show the percentage of germaria that contain the indicated number of UGCs for each genotype. *C587^{ts}*-mediated pupal-specific *tj dpp* or *tj en* double knocked down partially rescue the excess UGC induced by *C587^{ts}>tj RNAi*.

Figure S7

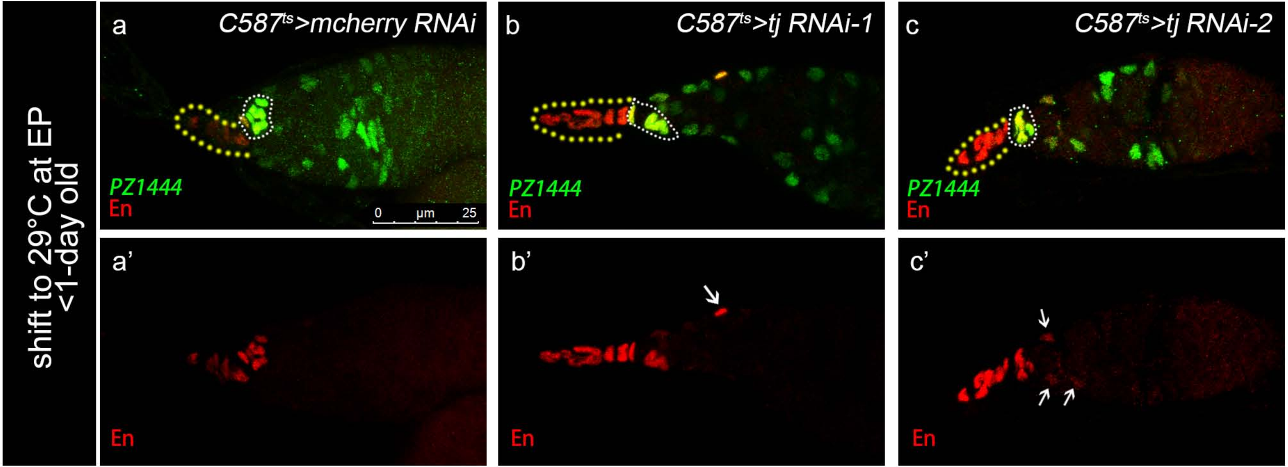
shift to 29°C at EP
<1-day old
a
C587ts>mcherry RNAi
PZ1444
En
0 μm 25
b
C587ts>tj RNAi-1
PZ1444
En
c
C587ts>tj RNAi-2
PZ1444
En
a'
En
b'
En
c'
En

Figure S7. ***C587^{ts}*****-mediated pupal-specific** ***tj*** **knockdown induces ectopic En in ECs.**

(a-c') Germaria are from females of indicated genotypes, which are maintained at 18°C till EP stage and then shifted to 29°C. The newly eclosed flies (<1-day old) are stained for En (red), to mark TF and CpCs. The *PZ1444/+* (marked by β-gal, red) is introduced to distinguish ECs from CpCs. Control group **(a, a')** exhibits specific expression of En in TF and CpCs. The *C587^{ts}>tj RNAi-1* **(b, b')** and *C587>tj RNAi-2* **(c, c')** groups exhibit ectopic En in ECs, as indicated by white arrows. Scale bar is shown in panel (a). Anterior is always to the left.

Figure S8

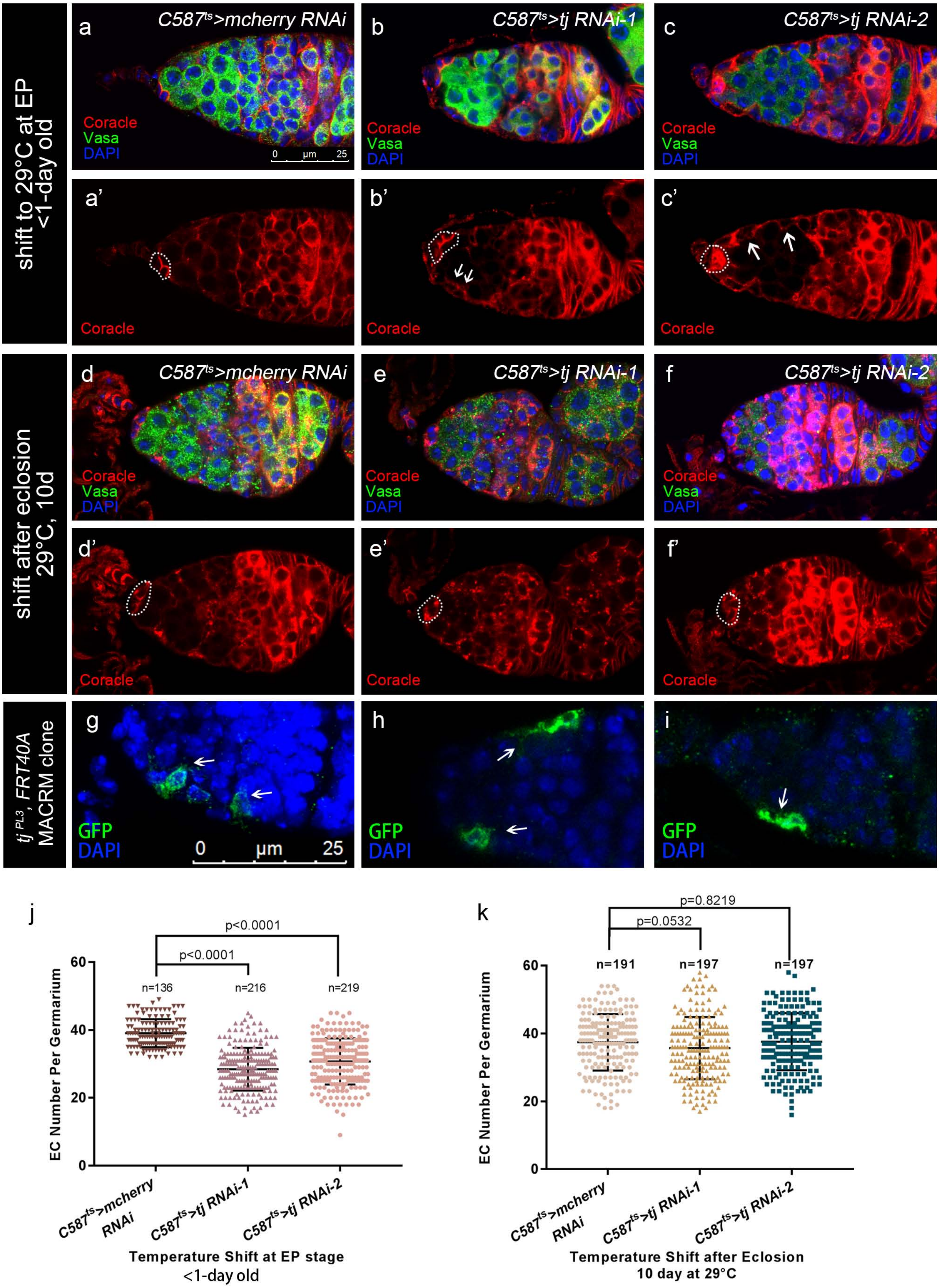

shift to 29°C at EP
<1-day old
shift after eclosion
29°C, 10d
tj PL3, FRT40A
MACRM clone
a
C587ts>mcherry RNAi
b
C587ts>tj RNAi-1
c
C587ts>tj RNAi-2
Coracle
Vasa
DAPI
0 μm 25
a'
b'
c'
d
e
f
d'
e'
f'
g
h
i
GFP
DAPI
j
p<0.0001
p<0.0001
n=136
n=216
n=219
EC Number Per Germarium
C587ts>mcherry RNAi
C587ts>tj RNAi-1
C587ts>tj RNAi-2
Temperature Shift at EP stage
<1-day old
k
p=0.8219
p=0.0532
n=191
n=197
n=197
Temperature Shift after Eclosion
10 day at 29°C

Figure S8. Tj regulates EC protrusions and EC number during pre-adult stage. **(a-f')** Germaria from females of indicated genotypes are stained for Vasa (green), to mark germline, Coracle (red), to mark cytoplasmic processes of the ECs. DAPI is blue. White broken ovals indicate CpCs. **(a-c')** Germaria from the newly eclosed flies (<1-day old), which are maintained at 18°C till EP stage and then shifted to 29°C, are examined. The control group **(a, a')** exhibits germline encapsulation by EC protrusions. Germaria of newly eclosed *$C587^{ts}$>tj RNAi-1* **(b, b')** and *$C587^{ts}$>tj RNAi-2* **(c, c')** flies, which are raised at 18 °C up to EP stage and then maintained at 29°C till eclosion, display loss of protrusions in some ECs. White arrows indicate ECs lack protrusions. **(d-f')** Germaria from females, which are raised at 18 °C up to eclosion and then shifted to 29°C for 10 days, are detected. The *$C587^{ts}$>tj RNAi-1* **(e, e')** and *$C587^{ts}$>tj RNAi-2* **(f, f')** groups exhibit normal EC protrusions, as the control group **(d, d')**. **(g-i)** More samples of *tj^{PL3}* mutant MARCM clone. The clonal ECs (green) exhibit impaired cellular protrusions. White arrows indicate short protrusions. **(j, k)** Graphs show EC number for each genotype as indicated. Pupal-specific knockdown of *tj* **(j)** results in reduced EC number compared with control group. Adult-specific knockdown of *tj* **(k)** does not cause loss of EC numbers. Error bars are shown as Means ± S.D. of each genotype. Scale bar for (a-f') is shown in panel (a). Scale bar for (i-k) is shown in panel (i). Anterior is always to the left.
